# *Who* Cares What Other People Think? A Longitudinal Investigation of the Role of Autonomy-Connectedness in Self-Esteem Change Trajectories and Instability

**DOI:** 10.1007/s10608-025-10604-2

**Published:** 2025-04-28

**Authors:** Laura E. Kunst, Marcel A. L. M. van Assen, Felix J. Clouth, Caroline Hunt, Maree J. Abbott, Joyce Maas, Marrie H. J. Bekker

**Affiliations:** 1 Department of Medical and Clinical Psychology, CoRPS - Center of Research on Psychological Disorders and Somatic Diseases, Tilburg University, Tias Building, Warandelaan 2, 5037 AB Tilburg, The Netherlands; 2 Mental Care Group, Mentaal Beter, Breda, The Netherlands; 3 Department of Methodology and Statistics, Tilburg University, Tilburg, The Netherlands; 4 Department of Sociology, Utrecht University, Utrecht, the Netherlands; 5 School of Psychology, The University of Sydney, Sydney, New South Wales Australia; 6 Center for Eating Disorders Helmond, Mental Health Center Region Oost-Brabant, Helmond, The Netherlands; 7 Department of Clinical Psychology, VU University, Amsterdam, The Netherlands; 8 RINO Amsterdam, Amsterdam, The Netherlands

**Keywords:** Self-esteem, Autonomy-connectedness, Latent growth mixture modeling, Anxiety, Depression

## Abstract

**Background:**

While low self-esteem is an established risk factor for depressive and anxiety disorders, psychological underpinnings of unstable self-esteem remain understudied. We investigated the role of autonomy-connectedness, the psychological capacity for self-governance, in self-esteem and its change trajectories and instability.

**Methods:**

Data spanning 12 years of the Longitudinal Internet studies for the Social Sciences [LISS] panel, a large, nationally representative population sample (*N* = 5367, ages 16–91), were used.

**Results:**

Mixed model analyses revealed that autonomy-connectedness was positively associated with self-esteem. Autonomy-connectedness component Sensitivity to others predicted less deterioration of self-esteem over time. Latent growth mixture modeling exploring individual differences revealed seven latent classes differing in slope and self-esteem instability over time. Individuals with healthy autonomy were more likely to belong to classes with above average self-esteem, but not necessarily more stable self-esteem trajectories. A subgroup (11%) displayed alarming autonomy deficits, which corresponded with low, stable self-esteem, as well as high levels of depressive and anxiety symptoms.

**Conclusions:**

Autonomous individuals have higher self-esteem and better mental health, but autonomy deficits were not necessarily associated with *unstable* self-esteem trajectories. Being sensitive to others buffers against declining self-esteem in older age. Results are discussed in light of depression and anxiety vulnerability.

**Supplementary Information:**

The online version contains supplementary material available at 10.1007/s10608-025-10604-2.

## Introduction

Self-esteem is one of the most widely studied concepts in social and clinical psychology and pertains to the subjective experience of one’s self-worth (Rosenberg, 1965). People with healthy self-esteem are more satisfied in their lives (Diener & Diener, 2009; Lyubomirsky et al., 2006), more resilient (Liu et al., 2014) and have more successful careers and higher work satisfaction (Salmela-Aro & Nurmi, 2007) than people with lower self-esteem. Low self-esteem inversely is related to mental health problems such as major depression and anxiety disorders (Keane & Loades, 2017). For depressive symptoms, low self-esteem acts as a vulnerability factor predicting subsequent depression, whereas for anxiety a reciprocal relation exists, in which low self-esteem predicts anxiety, but anxiety symptoms also leave a ‘scar’ of lowered self-esteem (Sowislo & Orth, 2013).

Although self-esteem is relatively stable in all age groups and in men as well as women (Kuster & Orth, 2013; Trzesniewski et al., 2003), it is not a fixed personality trait. Scientific interest has recently shifted to exploring self-esteem changes and instability within individuals (e.g., Hank & Baltes-Gotz, 2019; Okada, 2010; Sanchez-Queija et al., 2017). Intra-individual variations can be characterized as short-term, momentary situation-specific changes in ‘state’ self-esteem, often assessed in laboratory and experience sampling studies (e.g., Geukes et al., 2017; van Schie et al., 2019) and long-term trajectories of ‘trait’ self-esteem (e.g. Orth et al., 2018). Self-esteem instability scores can be computed for state as well as trait variables. Experience sampling studies often provide a relatively large number of observations, allowing computation of instability parameters (e.g., Geukes et al., 2017; Okada, 2010). In large, cohort studies with fewer observations, latent class analyses can be used to explore self-esteem instability (e.g., Morin et al., 2013; Mund & Neyer, 2016).

Laboratory and experience sampling studies have revealed that trait self-esteem and self-esteem instability are negatively and weakly related, suggesting that these constructs are connected, but distinct (Okada, 2010). Self-esteem instability is associated with depressive and anxiety symptoms (e.g., Crowe et al., 2019; van Tuijl et al., 2018) and prospectively predicts depressive symptoms, even when controlling for trait self-esteem (Sowislo et al., 2014). These findings suggest that not only one’s overall level, but also fluctuations in self-esteem relate to depression and anxiety vulnerability. Understanding what makes certain individuals prone to unstable self-esteem may elucidate the nature of this vulnerability.

Social theories of self-esteem stress that self-evaluations depend largely on how accepted people feel within their social environment (e.g. sociometer theory; Leary & Baumeister, 2000). One way of looking at self-esteem instability is that individuals who are highly focused on their social environment in terms of seeking guidance and approval, may experience frequent changes in self-esteem, because their self-evaluations are highly dependent on others. Inversely, having a strong ‘sense of self’ might protect one’s self-esteem against external influences.

Many theorists have sought to explain vulnerability for depression and anxiety by focusing on themes surrounding ‘sense of self’ and interpersonal in(ter)dependence. Beck (1983), for instance, developed the concept autonomy-sociotropy, in which ‘autonomy’ represented a strong focus on achievement and individualism, and sociotropy an excessive investment in interpersonal relationships. Beck (1983) theorized that autonomous versus sociotropic individuals would develop depression and anxiety following achievement-related and interpersonal stressors respectively (e.g., Rude & Burnham, 1993). This ‘congruence hypothesis’ received inconsistent empirical support, partly due to psychometric limitations of the ‘autonomy’ subscale (i.e., it did not measure ‘self-governance’; Coyne & Whiffen, 1995; Hmel & Pincus, 2002). Associations between sociotropy and depression and anxiety symptoms were fairly consistent and positive (Kunst et al., 2021), suggesting that investments in social relationships at the expense of oneself could be considered a vulnerability factor for anxiety and depression.

Whereas the autonomy-sociotropy construct focused on maladaptive aspects of social connectedness, Blatt (2008) considered interpersonal relatedness and self-definition as two equally important dimensions of personality development. He argued that humans occilate between the two, so that engagement in healthy relationships contributes to an evolved sense of self, which in turn promotes further connectedness, and so on. The corresponding ‘anaclitic’ forms of psychopathology are characterized by preoccupations with abandonment, rejection, dependency and loneliness, whereas ‘introjective’ psychopathology is characterized by an exaggerated focus on autonomy, self-definition and independence (Blatt, 2008). Gilbert’s (1989, 2000) social mentality theory differentiates between a care-seeking and care-giving mentality—reflecting a similar bipolarity in interaction with others.

Collectively, these theories stress the importance of an optimal balance between self-definition and ‘autonomy’ (as used by Beck, 1983) on one hand, and interpersonal relatedness on the other hand. Bekker (1993) and Bekker and van Assen (2006, 2008), in extension, argued that these dimensions should not be seen as ‘opposites’ and constructed a scale reflecting healthy autonomy (i.e., self-governance) under the assumption of interpersonal connectedness. Their concept of ‘autonomy-connectedness’ represents the capability to act upon one’s own, authentic needs and wishes, within a social context. This also implicates being aware of one’s social needs and being optimally sensitive to and influenced by others, without being overly dependent on them (Bekker, 1993; Bekker & van Assen, 2006).

Autonomy-connectedness consists of three interrelated components: (i) Self-awareness: being aware of one’s wishes, needs and opinions and the ability to express these in interpersonal interactions; (ii) Sensitivity to others’ (wishes, needs and opinions); and (iii) capacity for managing new situations: the ability to quickly feel at ease in novel situations and a tendency to explore (Bekker, 1993; Bekker & van Assen, 2006). Autonomy-connectedness has small to moderate associations with the Big Five personality traits (van Assen & Bekker, 2009) and psychological (in-)capacities such as assertiveness and alexithymia (Rutten et al., 2016) and therefore seems related to, but distinct from, various personality traits. Whereas other frameworks focus on ‘autonomy’ as a need to be met in particular contexts (i.e., how much autonomy employees are given at work; self-determination theory, Ryan & Deci, 2000), autonomy-connectedness measures the personal, general capacity for self-governance.

In line with ‘sociotropic’ and ‘anaclitic’ psychopathology formulations, individuals with anxiety and depressive symptoms have relatively low self-awareness and capacity for managing new situations, and high sensitivity to others (Bekker & Belt, 2006; Kunst et al., 2019; Rutten et al., 2016). This pattern reflects difficulty identifying and acting upon one’s authentic needs and wishes, and an excessive tendency to focus on needs, opinions and well-being of others. An advantage of the autonomy-connectedness scale is that it also assesses adaptive qualities of connectedness, in contrast to earlier autonomy formulations (see Bekker, 1993; Hmel & Pincus, 2002). Accordingly, individuals with antisocial personality traits were found to have lower sensitivity to others than healthy controls (Bekker & van Assen, 2017).

Following theories on autonomy, it seems likely that self-governance difficulties may be involved in low self-esteem, as vulnerability (preceding low self-esteem), scar (consequence) or reciprocal factor. Being unaware of and unable to act upon one’s needs may complicate healthy behaviors, such as enganging in reciprocal relationships and assertiveness, contributing to impaired needs fulfillment and lowered self-esteem. Having low self-esteem could, inversely, also complicate effective self-governance. Sociometer theory (Leary & Baumeister, 2000) and Beck’s (1983) formulations also suggest that autonomy deficits might contribute to *unstable* self-esteem, as ‘caring what people think’ could make individuals more vulnerable to changes in self-esteem following interpersonal events. In line with this reasoning, highly sociotropic individuals showed more decreases in self-esteem following social stressors than those low in sociotropy (Cikara & Girgus, 2010; Dasch et al., 2008). Laboratory-based studies also indicate that an underdeveloped sense of self is associated with pronounced self-esteem decreases following social stressors (Grondin et al., 2011; van Schie et al., 2018). However, the relation between autonomy-connectedness and self-esteem, its change trajectories and instability, have never been investigated. As autonomy-connectedness can be targeted in treatment (Maas et al., 2019), exploring associations between autonomy-connectedness and self-esteem instability could have important implications for the clinical management of depression and anxiety.

This study aimed to investigate associations between autonomy-connectedness and (i) overall trait self-esteem, (ii) linear self-esteem change trajectories, and (iii) individual differences in self-esteem change trajectories and instability. To complement earlier laboratory studies using small convenience samples, we used the large (*N* = 5367), nationally representative Longitudinal Internet Studies for the Social sciences (LISS) panel (Tilburg University, the Netherlands). Assessing self-esteem ‘instability’ is somewhat complicated in cohort studies due to a limited number of measurements. We therefore approximated ‘instability’ by assessing linear changes over time on a population sample level using linear mixed models; as individual differences in (random) intercept and slope parameters in linear mixed models; and as change trajectories and instability parameters in latent growth mixture modeling (LGMM).

In addition to assessing instability, LGMM also illustrates individual differences in change trajectories and instability. Several recent studies in adolescents and young adults used LGMM to identify distinct change trajectories in self-esteem over multiple years. For instance, Morin et al., (2013; over a 4-year period) and Mund and Neyer (2016; over a 5-year period) identified a total of four latent classes, with a high and stable self-esteem class (29% in Mund & Neyer, 2016, 13.5% in Morin et al., 2013), an increasing and stabilizing class (15.1% and 11.0%, respectively), a moderate and relatively unstable class (31.7%; 56.2%), and a low and highly unstable class which decreased slightly over time (24.2%; 19.3%). Poorer mental health and relationship quality were found for participants in the classes with unstable self-esteem patterns, even in the presence of moderate overall levels of self-esteem (Mund & Neyer, 2016). However, as self-esteem has been shown to follow age-specific trajectories (increases from childhood to young adulthood, further increases until the age of 50–60, and then gradual decreases; Orth et al., 2018), it is important to study samples including a wider age range. Additionally, the psychological underpinnings of these latent classes remain to be clarified.

In sum, self-esteem and its instability seem important risk factors for depressive and anxiety symptoms, and understanding the role of autonomy-connectedness in trait self-esteem, its change trajectories and instability, may aid clinical management of self-esteem related psychopathology. In the present study, we expected positive associations between self-esteem and autonomy-connectedness components self-awareness and capacity for managing new situations, and negative associations with sensitivity to others. Associations between autonomy-connectedness and linear trajectories (i.e., decreasing or increasing trends) in self-esteem over time were explored. Finally, individuals with autonomy-connectedness deficits (i.e., low self-awareness and capacity for managing new situations and high sensitivity to others) were expected to have more unstable self-esteem trajectories than individuals with healthier autonomy patterns, due to an increased sensitivity to their social environment and more difficulty acting upon one’s needs. Demographic characteristics, anxiety and depressive symptoms and socially desirable reporting style were also examined in light of the study aims.

## Method

### Design, Procedure and Respondents

The present paper made use of data of the LISS (Longitudinal Internet studies for the Social Sciences) panel administered by CentERdata (Tilburg University, the Netherlands). The LISS panel is a representative sample of the Dutch population aged 16 or older, who complete monthly surveys on social, economic and health related topics. The sample is based on a true probability sample of households drawn from the population register. All respondents provided informed consent. If respondents had no access to a computer with internet, they were provided with one (more information on data collection is available on www.lissdata.nl; our analysis code can be accessed through 10.34894/4NDGHW).

**Fig. 1:**
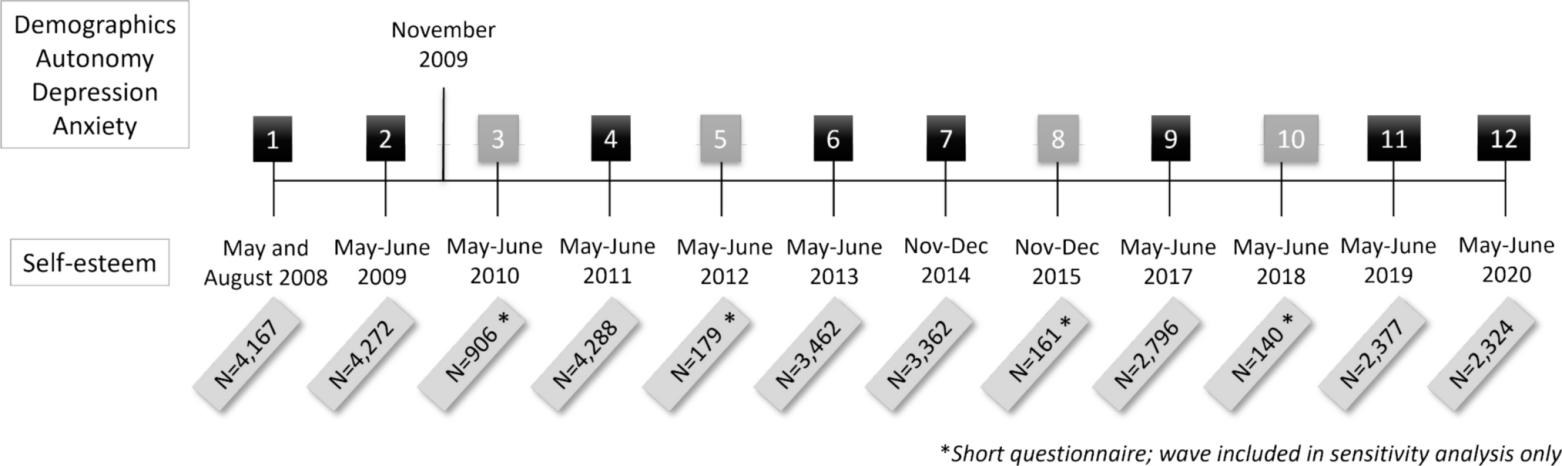
Overview of the Longitudinal Internet Studies for the Social Sciences (LISS) Waves (1–12) for Self-Esteem, Autonomy-Connectedness, Depression and Anxiety (N = 5367)

The LISS dataset contains 12 waves administered between 2008 and 2020 (Fig. 1). Autonomy-connectedness was measured once (in 2009) and was used to predict change trajectories in self-esteem, which had measurements spanning from 2008 through 2020. Self-esteem was usually assessed in May and questionnaires were repeated in June for non-responders. Exceptions include the baseline wave, which was repeated for non-responders in August, and wave 7 and 8, which were administered in November (non-responders in December). In waves 3, 5, 8 and 10, respondents who completed the questionnaires in the preceding year were offered a shorter questionnaire, which did not contain the self-esteem measure, providing lower response rates. Autonomy-connectedness, depressive and anxiety symptoms were administered as an extra questionnaire in November 2009. Demographics were also derived from the 2009 measurement and social desirability was assessed in 2008.

Respondents were included in the dataset if they had completed the 2009 autonomy-connectedness questionnaires and at least one self-esteem measure, providing a total of 5367 respondents (see Table 1). A total of 28,434 (44.15%) self-esteem measurements were completed, out of the total possible 64,404 (5367 respondents × 12 waves). As respondents could not partly complete the self-esteem and autonomy-connectedness questionnaires, they did not have missing values. Missing self-esteem waves were not replaced or imputed, because both statistical approaches (see Statistical analysis) are based on full information maximum likelihood (FIML), which accommodates time-varying covariates and can deal with missing values (e.g., Polit & Gillespie, 2010). Imputing missing values for the waves with few responses would also be invalid, as four waves (3, 5, 8 and 10) had between 83 and 97% observations missing and missing values were systematic (not random) due to the process of data collection in the LISS panel. See 10.34894/4NDGHW for the syntax files used for data analysis (Table 1).

**Table 1:** Sociodemographic Characteristics of Respondents in 2009 (N = 5367)

	Mean or *N*	*SD* or %
Female	2855	53.2
Age	48.83 (range 16–91)	17.06
Net monthly income of the household (€)	3022.65	6427.72
Highest completed education		
Primary school	526	9.8
Intermediate secondary education	1465	27.3
Higher secondary education	613	11.4
Intermediate vocational education	1148	21.4
Higher vocational education	1193	22.2
University degree	405	7.5
Missing	17	0.3

### Measures

#### Self-Esteem

Self-esteem was assessed using the 10-item Rosenberg Self-Esteem Scale (RSES; Rosenberg, 1965). The RSES is a widely used instrument that measures positive and negative self-evaluations (e.g., ‘I feel that I'm a person of worth, at least on an equal plane with others’). The original as well as Dutch RSES have been shown to have excellent psychometric properties (Franck et al., 2008; Sinclair et al., 2010). In the LISS panel items were scored on a 7-point Likert scale ranging from 1 (totally disagree) to 7 (totally agree) instead of the original 4-point scale. The scores were averaged for analysis. In the present study, internal consistency was good, with Cronbach’s alphas ranging from *α* = 0.89 (Wave 1) to *α* = 0.92 (Wave 8).

#### Autonomy-Connectedness

Autonomy-connectedness was measured once (in 2009), using the 30-item Autonomy-Connectedness Scale (ACS-30; Bekker, 1993; Bekker & van Assen, 2006). The ACS-30 consists of three subscales representing the three autonomy-connectedness components Self-awareness (e.g., ‘If I am asked what I want, I mostly know the answer immediately’), Sensitivity to others (e.g., ‘If I have things my own way against the will of others, I usually get very restless’), and Capacity for managing new situations (e.g., ‘I easily come to grips with a new problem on my own’). All items can be rated on a scale ranging from 1 (‘disagree’) to 5 (‘agree’) and averaged scores are used for analysis. The ACS-30 has been found to have good reliability and construct validity (Bekker & van Assen, 2006). In this study, internal consistency was good, with Cronbach’s *α* = 0.75 for Self-awareness, *α* = 0.78 for Sensitivity to others, and* α* = 0.74 for Capacity for managing new situations.

#### Depressive Symptoms

Depressive symptoms were measured using the Beck Depression Inventory (BDI-II-NL; Beck et al., 1996), a well-known instrument with excellent psychometric properties (Beck et al., 1988, 2002). Its 21 items consist of depressive symptoms (e.g., anhedonia, guilt) rated on a scale from 0 to 3 (total scores range from 0 to 63). Internal consistency in the present study was good, Cronbach’s *α* = 0.85.

#### Anxiety Symptoms

Anxiety symptoms were assessed using the 10-item Anxiety subscale of the Symptom Checklist (SCL-90). The SCL-90 assesses a broad range of psychopathology symptoms (Arrindell & Ettema, 1975; 2005; Derogatis, 1994). Respondents indicate on a 5-point Likert scale the extent to which they experienced symptoms in the past week (e.g., feeling tense). Anxiety subscale scores range from 10 to 50 and its reliability in the present study was Cronbach’s *α* = 0.91.

#### Social Desirability

Social desirability was assessed at baseline (2008) using a 10-item version of the Crowne-Marlowe Social Desirability Scale (Crowne & Marlowe, 1960; Fischer & Fick, 1989). The scale contains items on excessively desirable (e.g., “I never hesitate to go out of my way to help someone in trouble”) and ‘embarrassing’ behavior (e.g., “I can remember ‘playing sick’ to get out of something”). Respondents rate statements as ‘true’ or ‘false’, and depending on the statement, items are scored as 0 (not socially desirable) or 1 (socially desirable). High total scores represent higher social desirability tendency. Reliability in this study was relatively poor, Cronbach’s *α* = 0.52.

### Statistical Analysis

All analyses were two-tailed. We used an alpha of 0.001 because statistical power of detecting small true effect sizes still approximates 1 due to the very large sample size, and we wanted to limit the type I error rate. Because of very low completion rates at waves 3, 5, 8 and 10, analyses were carried out using only the remaining 8 waves (colored black in Fig. 1), but all analyses were repeated using all timepoints as sensitivity analyses (see Online Appendix A). Means, standard deviations and correlations of variables under study were computed as descriptive statistics.

Data were analyzed using two complementary statistical techniques answering different research questions. Using linear mixed models (LMM) we tested hypotheses on associations between autonomy-connectedness components and self-esteem in the population sample; we tested individual differences in the linear trajectories of self-esteem over time; and we assessed whether possible differences therein could be explained by autonomy-connectedness. Using latent growth mixture modeling (LGMM) we set out to further explore and illustrate individual differences in self-esteem change trajectories and instability; and we tested whether obtained classes differed with respect to personal characteristics, including autonomy-connectedness. Hence, LMM focused on identifying and explaining linear trajectories of self-esteem over time in the sample as a whole, whereas LGMM built on the LMM by further exploring and illustrating classes with different self-esteem trajectories.

#### Linear Mixed Models

Longitudinal LMM was conducted in SPSS v26. In the first analyses we estimated the intraclass correlation (ICC) and fitted the heterogeneous first-order autoregressive (ARH1) structure to the covariance matrix in a model with a linear effect of time on self-esteem. Then we tested the random effect of time to examine if individuals differed in their self-esteem trajectories over time. All these models were estimated and tested using restricted maximum likelihood (REML), as REML is more appropriate when testing random effects (Heck et al., 2021). The latter model formed our base model (Model 1) for the subsequent analyses and included 14 parameters (two fixed effect parameters for intercept and effect of time, and 12 for the random effects: intercept, slope, and their covariance, and eight variances and one autoregressive correlation for the ARH1 covariance structure). The effect size for time was calculated by dividing changes in self-esteem over time by the baseline standard deviation of self-esteem (2008; Glass’s *d*; Lakens, 2013).

To evaluate the predictive role of autonomy-connectedness (measured once, in 2009) on self-esteem trajectories, three models with increasing complexity were estimated using maximum likelihood (ML), which is recommended when testing fixed effects (Heck et al., 2021). Model 2 included the three autonomy-connectedness components as fixed effects, where each component was centered. Model 3 added the three interaction terms between centered autonomy-connectedness components and time. Model 4 displays the final model, excluding the irrelevant interaction terms. To facilitate interpretation of any significant interaction terms, simple slope analyses were performed. Models were compared to each other using the -2LL statistic and the Bayesian information criterion (BIC). Random effects parameters were tested with the Wald *z*-test, whereas fixed effect parameters were tested using the *t*-test.

Three sensitivity analyses were conducted, all with Model 4 as starting point and increasing data or model complexity in three steps. First, Model 4 was estimated using all data (i.e., of all 12 timepoints, instead of 8). Second, we controlled for effects of age, sex, net household income and education (categorical), which were added to Model 4 (using the original 8 timepoints) as fixed covariates (adding 8 parameters). Third, in addition to the other control variables, we also added (centered) depressive and anxiety symptoms and their interactions with time to Model 4 (also using the original 8 timepoints), to verify whether linear changes in self-esteem were also predicted by depressive and anxiety symptoms, and whether the results on autonomy-connectedness were maintained. The full sensitivity analyses are reported in Online Appendix A. See 10.34894/4NDGHW for the code of our mixed model analyses in SPSS.

#### Latent Growth Mixture Modelling

LGMM was conducted to identify classes of individuals with similar trajectories of self-esteem over time, to further explore individual differences (Muthén & Muthén, 2000; Ram & Grimm, 2009). Models with one to ten latent classes were estimated with time entered into the model as a linear predictor. Sensitivity analyses with time not restricted in its functional form were performed, but did not suggest a non-linear relation (see Online Appendix A). For each model, random effects for the class intercepts and fixed effects for the slopes were estimated where residual variances were allowed to vary across latent classes. A class specific mean of the intercept represents the initial level of self-esteem at baseline (2008) for that class and the random effect represents the class-specific inter-individual variation of the intercept (Mund & Neyer, 2016). The variance of the class-specific random effects and residuals together determine the relative inter-individual stability of the trends of self-esteem in each class, which can be interpreted as a class-specific ICC.

One issue with LGMM is its proneness to converge to local maxima. To avoid this, each model was estimated with 200 random sets of start values. Latent Gold (Vermunt & Magidson, 2016) uses a combination of the Expectation-Maximation (EM; Dempster et al., 1977) algorithm and a Newton–Raphson algorithm (Haberman, 1988) to optimize the model log-likelihood function. Our final model converged within 210 EM and 8 Newton–Raphson iterations. To determine the optimal number of classes, following recommendations by Jung and Wickrama (2008) and van de Schoot et al. (2017), model fit indices such as the BIC (Schwarz, 1978), Akaike information criterion (AIC; Akaike, 1974), and consistent Akaike information criterion (CAIC; Anderson et al., 1998) were compared. Preferably, the bootstrap likelihood ratio test (BLRT; McLachlan & Peel, 2004) should be consulted as well, however, due to the complexity of our model including up to 10 classes, such Mont-Carlo based resampling method was infeasible. As these model selection tools are sensitive to large sample sizes (van de Schoot et al., 2017) as is the case in this study, interpretability and size of the classes were taken into account when deciding on the optimal number of classes. We were specifically interested in classes that show a decreasing or increasing trend of self-esteem over time, but not interested in classes representing a very small part of the sample (say less than 1%).

In a last step, the latent classes were compared with respect to their mean level of autonomy-connectedness (measured in 2009), demographic characteristics (age, sex, net monthly household income and education; 2009), and mental health indices (anxiety and depressive symptoms; 2009) using the bias-adjusted three-step approach (Bakk et al., 2013; Vermunt, 2010). This approach involves performing an ANOVA that is weighted with the inverse of the classification error probabilities. Incorporating these weights is necessary to take uncertainty in the classification into account correctly. We used the pseudo *R*^*2*^ as effect size measure comparable to eta squared in ANOVA. The pseudo *R*^*2*^ is based on the mean squared prediction error and represents the proportional reduction of errors in the estimated model compared to the baseline model without predictors (Vermunt & Magidson, 2016). Glass’s *d* effect sizes (Lakens, 2013) were computed for outcome variables, comparing each class to the overall mean. As an additional analysis suggested during manuscript review, social desirability was also compared across classes. LGMM analyses were performed in SPSS version 26 and Latent Gold version 5.1.0.20227 (Vermunt & Magidson, 2016). See 10.34894/4NDGHW for the Latent Gold code of our analyses.

## Results

Descriptive statistics are depicted in Table 2.

**Table 2:** Means, Standard Deviations and Correlations Between Variables Under Study

	Means		Correlations							
	*M*	*SD*	1	2	3	4	5	6	7	8
1. Age	48.83	17.06	–							
2. Female sex	–	–	-0.059	–						
3. Baseline self-esteem	5.66	0.97	0.13	− 0.082	-					
4. Self-awareness	3.81	0.69	0.11	− 0.14	0.44	–				
5. Sensitivity to others	3.24	0.53	− 0.053	0.33	− 0.22	− 0.33	–			
6. Capacity for managing new situations	3.06	0.81	− 0.016^*a*^	− 0.15	0.33	0.41	− 0.31	–		
7. Depressive symptoms	5.48	5.70	0.094	0.12	− 0.38	− 0.31	0.23	− 0.31	–	
8. Anxiety symptoms	13.66	5.12	− 0.12	0.11	− 0.35	− 0.30	0.28	− 0.26	0.61	
9. Social desirability	5.93	1.97	0.22	0.02^*b*^	0.21	0.11	− 0.068	0.084	− 0.14	− 0.18

Sex is coded as 0 = male, 1 = female. All correlations are significant (*p* < 0.001), except ^a^ (*p* = 0.252) and ^b^ (*p* = 0.199). Demographics, Autonomy-Connectedness, Depression and Anxiety were assessed in 2009; baseline Self-Esteem and Social Desirability in 2008.

### Preparatory Mixed Model Analyses

The ICC of 0.70 indicated that most variation in self-esteem scores stemmed from differences between people rather than from intra-individual changes in self-esteem (variation within their own scores). Incorporating the ARH1 covariance structure improved model fit, *χ*^2^(8) = 1,113.56, *p* < 0.001. Adding a random effect of time to the latter model further improved the fit, *χ*^2^(2) = 334.61, *p* < 0.001, showing that there were individual differences in (linear) trajectories of self-esteem over time. In the latter model, error variances generally decreased over time, from values equal to 0.318 at wave 1 (2008) to 0.186 at wave 12 (2020). The estimated autocorrelation was 0.143 (Wald *z* = 11.65, *p* < 0.001). The variances of random intercept and slope were 0.657 (*z* = 37.68, *p* < 0.001) and 0.287 (*z* = 15.22, *p* < 0.001), respectively, and were negatively correlated (*r* = − 0.156, Wald *z* = − 4.66, *p* < 0.001). The negative correlation means that respondents with higher baseline self-esteem tended to have lower (less positive or more negative) slopes, indicative of a lower increase or a higher decrease of self-esteem over time, compared to people with lower baseline self-esteem.

### Mixed Models Results

Table 3 shows the fixed-effect estimates, variances, intercept and slope and their covariance, and fit indices of models 1–4. On average, self-esteem decreased slightly from 2008 to 2020 (− 0.079 points per 10 years, *d* = -0.081, small effect; Table 3, Model 1), suggesting a largely stable average trajectory on population level. Upon adding the autonomy-connectedness components to Model 2, model fit improved compared to Model 1, *χ*^2^(3) = 1766.20, *p* < 0.001, Table 3. Partly in line with the hypotheses, Self-awareness and Capacity for managing new situations had positive associations with self-esteem (Table 3, Model 2), whereas Sensitivity to others appeared to be unrelated to self-esteem. Thus, individuals with low Self-awareness and Capacity for managing new situations, seem to have poorer self-esteem, whereas for Sensitivity to others, the association seems more complex.

**Table 3:** Mixed Model Analyses on the Predictive Effects of Time and Autonomy-Connectedness on Self-Esteem (*N* = 5171)

*Fixed effects*	Model 1			Model 2			Model 3			Model 4		
	*B*	*SE B*	*p*	*B*	*SE B*	*p*	*B*	*SE B*	*p*	*B*	*SE B*	*p*
Intercept	5.62	0.013	< 0.001	5.62	0.11	< 0.001	5.62	0.11	< 0.001	5.62	0.11	< 0.001
Time	− 0.079	0.014	< 0.001	− 0.080	0.014	< 0.001	− 0.079	0.14	< 0.001	− 0.080	0.014	< 0.001
Self-awareness				0.53	0.017	< 0.001	0.54	0.018	< 0.001	0.53	0.017	< 0.001
Sensitivity to others				− 0.060	0.021	0.004	− 0.082	0.022	< 0.001	− 0.092	0.022	< 0.001
Capacity for managing new situations				0.19	0.014	< 0.001	0.20	0.015	< 0.001	0.19	0.014	< 0.001
Self-awareness × Time							− 0.047	0.023	0.037			
Sensitivity to others × Time							0.073	0.028	0.009	0.10	0.026	< 0.001
Capacity for managing new situations × Time							− 0.019	0.019	0.322			
*Random effects*												
Intercept variance	0.66			0.42			0.42			0.42		
Slope (time) variance	0.29			0.29			0.28			0.28		
Intercept-time covariance	− 0.068			− 0.040			− 0.038			− 0.039		
*Model fit indices*												
	*-2LL* = 54,442.24 *BIC* = 54,585.11			*-2LL* = 52,676.04 *χ*^*2*^(3) = 1,766.20, *p* < 0.001 *BIC* = 52,849.53			*-2LL* = 52,651.88 *χ*^*2*^(3) = 24.16, *p* < 0.001 *BIC* = 52,855.98			*-2LL* = 52,659.67 *χ*^*2*^(1) = 16.37, *p* < 0.001 *BIC* = 52,843.36		
Number of parameters	14			17			20			18		

Unit of time = per 10 years. -2LL for both model 3 and 4 were compared to model 2

Adding the three interactions of autonomy-connectedness with time again improved model fit (*χ*^2^(3) = 24.16, *p* < 0.001; Table 3, comparing Model 3 to Model 2), but Model 3’s BIC suggested worse fit than Model 2. As Model 3 indicated that Self-awareness and Capacity for managing new situations did not predict self-esteem trajectory over time, we fitted our final Model 4 with all three main effects and only the interaction of time with Sensitivity to others. Model 4 showed improved model fit compared to Model 2 (*χ*^2^(1) = 16.37, *p* < 0.001; *BIC* = 52,843.36), with a positive interaction effect (*B* = 0.10, *SE* = 0.026, *t*(3,240.44) = 4.051, *p* < 0.001). Simple slope analyses showed that self-esteem did not change over time for people high in Sensitivity to others (for + 1SD: slope = − 0.025, *SE* = 0.019, *t*(3,280.92) = − 1.28, *p* = 0.201), whereas self-esteem decreased for people with average (slope = − 0.080, *SE* = 0.014, *t*(3,249.37) = -5.80, *p* < 0.001), and low Sensitivity to others (for -1SD: slope = − 0.13, *SE* = 0.019, *t*(3,209.17) =  − 7.00, *p* < 0.001). In summary, Self-awareness and Capacity for managing new situations were unrelated to self-esteem development over time for the sample population as a whole. Individuals with high Sensitivity to others reported less decrease in self-esteem over time than those with lower Sensitivity to others.

Sensitivity analyses of the mixed models analyses are reported in Online Appendix A. Using all 12 waves instead of 8 did not alter the results (Online Appendix A). Adding demographic characteristics age, sex, net income of the household and education level to Model 4 improved model fit (− *2LL* = 48,502.95, *χ*^2^(8) = 4,156.72, *p* < 0.001; *BIC* = 48,766.30). Age was positively associated with self-esteem, *B* = 0.0045, *SE* = 0.00064, *p* < 0.001, suggesting that older individuals had higher self-esteem. Older individuals however also showed more decline in self-esteem over time (time x age interaction: *B* = − 0.0041, *SE* = 0.00092, *p* < 0.001). Having completed primary education only was associated with lower self-esteem than having a university degree (*B* = − 0.23, *SE* = 0.051, *p* < 0.001; Online Appendix A). However, addition of these demographic characteristics did not change the findings on autonomy-connectedness.

As a final sensitivity analysis, depressive and anxiety symptoms (measured in 2009) and their interactions with time were added to Model 4. This model again showed improved fit, *− 2LL* = 47,868.18, *χ*^2^(2) = 634.77, *p* < 0.001; *BIC* = 48,151.78, with both depressive (*B* = − 0.039, *SE* = 0.0023, *p* < 0.001) and anxiety (*B* = -0.016, *SE* = 0.0025, *p* < 0.001) symptoms being negatively associated with self-esteem. Main results on effects of autonomy-connectedness on Self-esteem were unaffected, that is, Self-awareness (*B* = 0.42, *SE* = 0.016, *p* < 0.001) and Capacity for managing new situations (*B* = 0.13, *SE* = 0.014, *p* < 0.001) still predicted self-esteem, and there was a positive interaction effect between Sensitivity to others and time (*B* = 0.10, *SE* = 0.027, *p* < 0.001). No interactions between depressive symptoms and time, and anxiety symptoms and time were found (see Online Appendix A).

The random effects in the mixed models analyses (see Table 3) suggest individual differences in baseline self-esteem as well as change trajectories, which were partly explained by effects of autonomy-connectedness, demographics, and depressive and anxiety symptoms. These individuals differences and self-esteem instability were further explored using LGMM.

### LGMM Model Selection

Table 4 summarizes the model fit indices for the 1 to 10 class solutions for LGMM, with the 10-class solution providing the best fit. The 7-class solution was selected because classes 8 through 10 did not provide additional substantive information compared to classes 1 through 7 and yielded very small classes. More specifically, the 8-class solution resulted in a class that contained less than 1% of all cases, and the 9- and 10-class solutions further split up the high and stable self-esteem classes, not contributing to an enhanced understanding of individual differences in self-esteem trajectories.

**Table 4:** Model Fit Indices for 1 to 10 Class Solutions

Classes	LL	BIC	aBIC	AIC	CAIC
1	− 28,146.3	56,326.9	56,314.2	56,300.5	56,330.9
2	− 25,691.7	51,460.7	51,432.1	51,401.4	51,469.7
3	− 25,289.1	50,698.5	50,654.0	50,606.3	50,712.5
4	− 24,964.2	50,091.6	50,031.2	49,966.4	50,110.6
5	− 24,714.5	49,635.1	49,558.8	49,476.9	49,659.1
6	− 24,563.4	49,375.9	49,283.8	49,184.9	49,404.9
7	− 24,453.7	49,199.4	49,091.3	48,975.4	49,233.4
8	− 24,337.6	49,010.1	48,886.2	48,753.2	49,049.1
9	− 24,283.3	48,944.5	48,804.7	48,654.6	48,988.5
10	− 24,225.3	48,871.3	48,715.6	48,548.5	48,920.3

aBIC, sample-size adjusted BIC; AIC, Akaike information criterion; BIC, Bayesian information criterion; CAIC, Consistent Akaike information criterion; LL, Log Likelihood

### Latent Class Characteristics

**Fig. 2:**
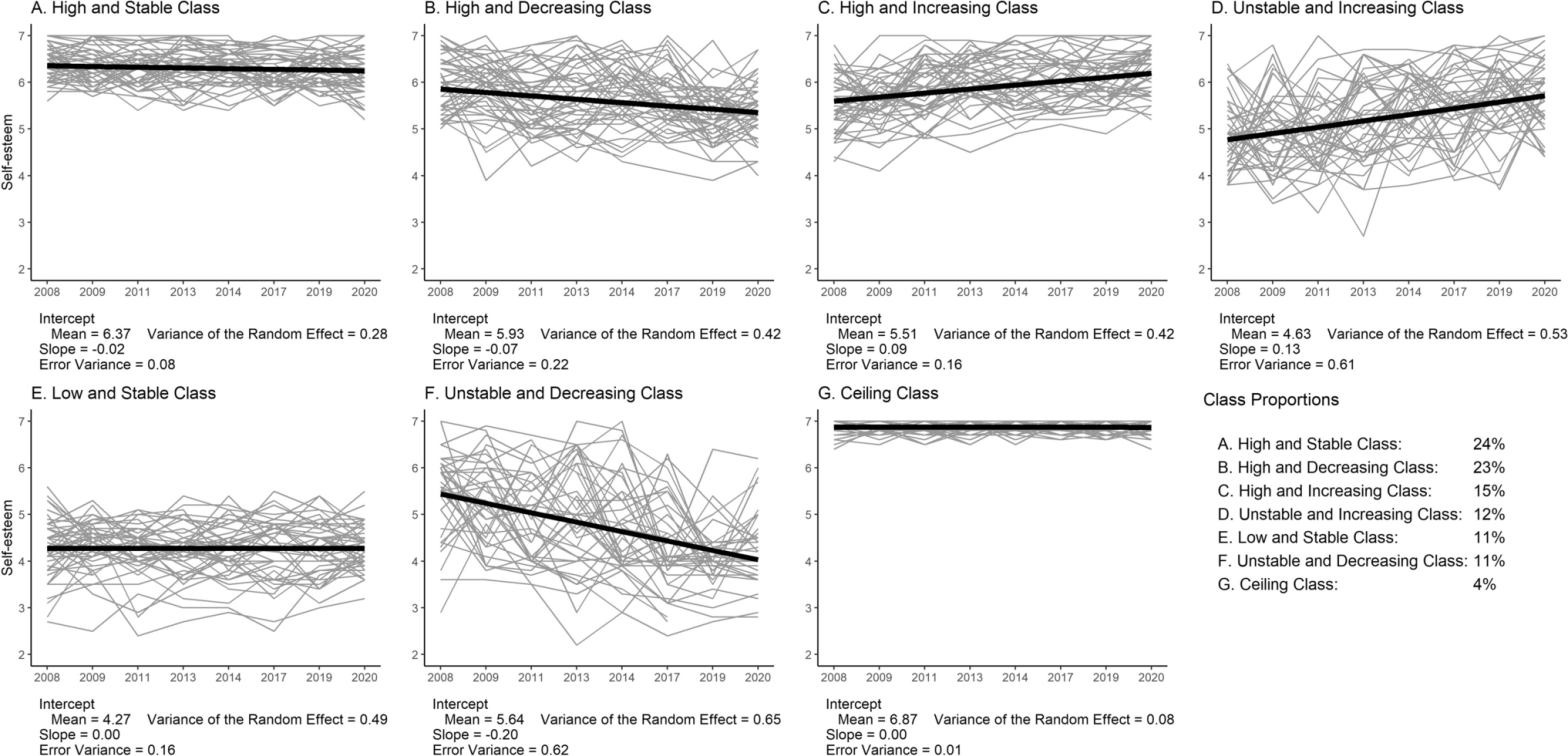
Change Trajectories in Self-Esteem over Time (Waves 1, 2, 4, 6, 7, 9, 11, 12) of 50 Randomly Selected Participants Per Class (Grey), Including Their Mean Trajectory (Black)

Figure 2 displays self-esteem trajectories of 50 randomly selected respondents per latent class. Table 5 displays the class parameters and autonomy-connectedness, clinical correlates and demographic characteristics across latent classes, including 99.9% confidence intervals of class averages on these variables and their (Pseudo) *R*^*2*^ values. Note that class membership had a large effect on Self-awareness, Capacity for managing new situations, anxiety- and depressive symptoms, (*R*^*2*^ ≥ 0.16), medium effects on Sensitivity to others and age (*R*^*2*^ between 0.06-0.08), and hardly an effect on income (*R*^*2*^ < 0.01). As income hardly differed across classes, we focus on the other variables for interpreting the classes.

**Table 5:**
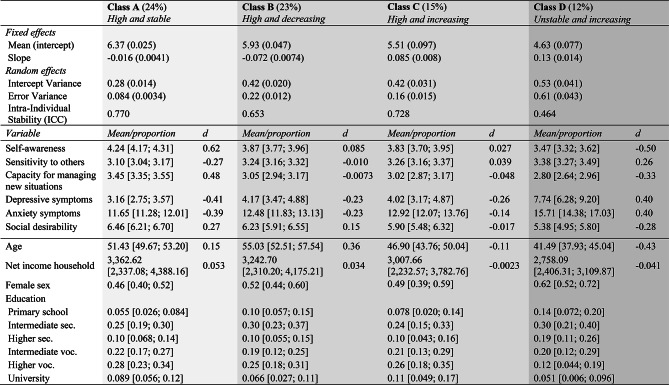

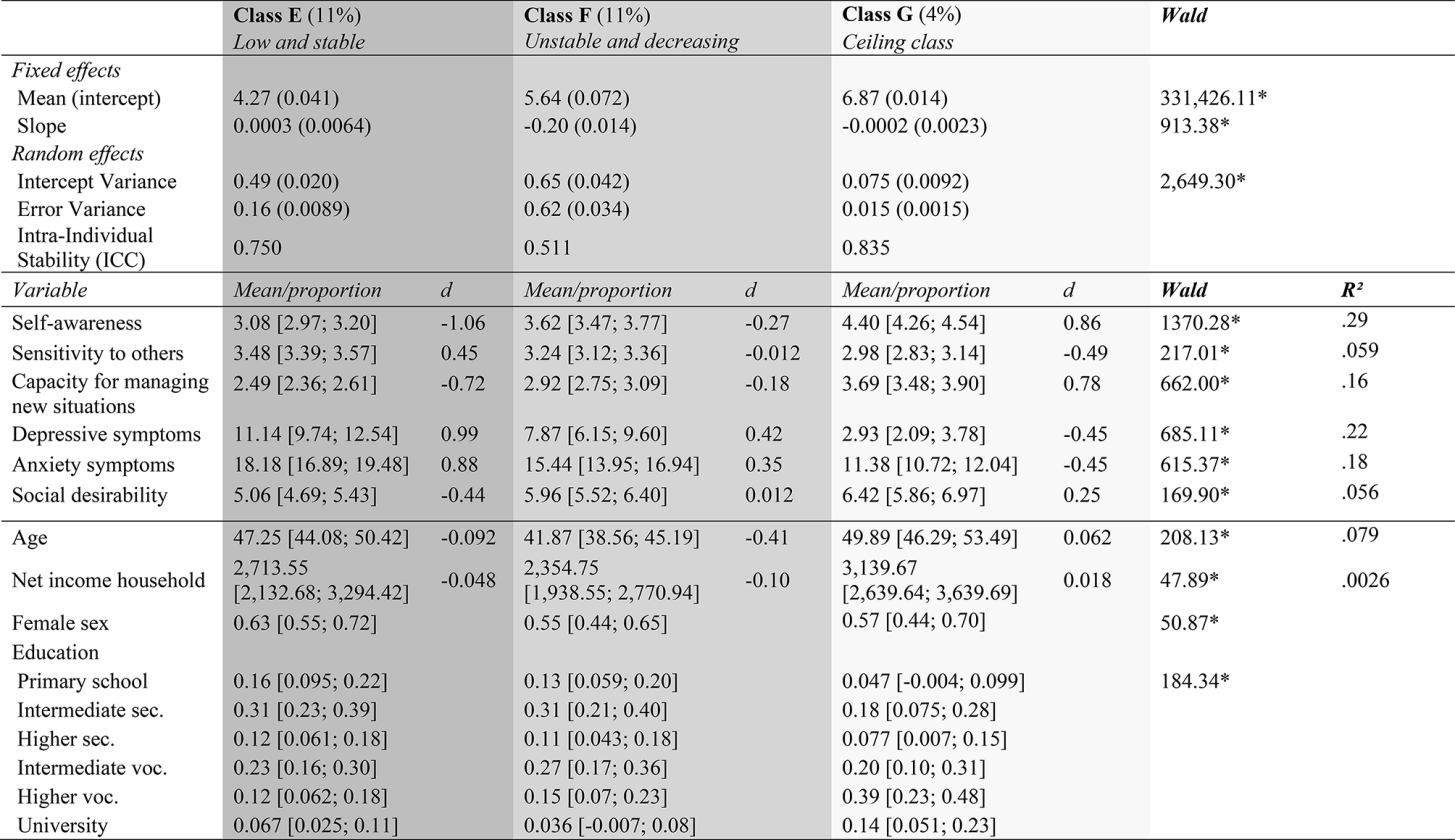
Model Parameters (Standard Error), Demographic Characteristics, Autonomy-Connectedness and Clinical Correlates of Latent Classes

The numbers between brackets represent 99.9% confidence intervals for the estimated class means or proportions. For Sex and Education, proportions are presented. The reference category for Education is primary education only, sec = secondary, voc = vocational. Lighter shades of grey represent higher levels of wellbeing, either in terms of self-esteem, autonomy-connectedness or mental health. The pseudo R^2^ indicates the proportion of variance explained by the latent class structure. *p < 0.001.

Classes A and G (lightest grey, together 28%) had the highest baseline self-esteem (6.37 and 6.87, respectively), were most stable (ICCs of 0.77 and 0.84), and showed no (decreasing or increasing) trend. Both classes were characterized by higher than average autonomy (i.e., higher Self-awareness, *d*_A_ = 0.62 and *d*_G_ = 0.86; lower Sensitivity to others, *d*_A_ = -0.27 and *d*_G_ = -0.49; higher Capacity for managing new situations, *d*_A_ = 0.48 and *d*_G_ = 0.78), as well as less depressive (*d*_A_ = − 0.41 and *d*_G_ = -0.45) and anxiety symptoms (*d*_A_ = -0.39 and *d*_G_ = − 0.45). People in these classes were relatively older (*d*_A_ = 0.15 and *d*_G_ = 0.062) and well-educated. Neither sex was overrepresented in these classes.

Classes B and C (light grey, together 38%) had average to above average baseline self-esteem (5.93 and 5.51, respectively), but were less stable (ICCs of 0.65 and 0.73), as Class B showed a small negative trend over time (*b* = − 0.072), and Class C had a small positive trend (*b* = 0.085). Their autonomy-connectedness was close to average (i.e., Self-awareness, *d*_B_ = 0.085 and *d*_C_ = 0.027; Sensitivity to others, *d*_B_ = − 0.010 and *d*_C_ = 0.039; Capacity for managing new situations, *d*_B_ = -0.0073 and *d*_C_ = − 0.048), with below average depressive (*d*_B_ = -0.23 and *d*_C_ = − 0.26) and anxiety (*d*_B_ = − 0.23 and *d*_C_ = − 0.14) symptoms. Individuals in Class B were older than in other classes (*d*_B_ = 0.36), whereas individuals in Class C were younger (*d*_C_ = − 0.11). Neither sex was overrepresented in these classes.

Classes D and F (darker grey, together 22%) were characterized by highest instability (ICCs 0.46 and 0.51). Whereas Class D was characterized by low baseline self-esteem (4.63) that increased over time (*b* = 0.13), Class F had average self-esteem at baseline (5.64), which decreased over time (*b* = − 0.20). Both classes were characterized by some autonomy deficits (measured in 2009), which were more severe in Class D than in Class F (i.e., Self-awareness, *d*_D_ = − 0.50 and *d*_F_ = − 0.27; Sensitivity to others, *d*_D_ = 0.26 and *d*_F_ = − 0.012; Capacity for managing new situations, *d*_D_ = − 0.33 and *d*_F_ = -0.18). Both classes had above average depressive (*d*_D_ = 0.40 and *d*_F_ = 0.42) and anxiety (*d*_D_ = 0.40 and *d*_F_ = 0.35) symptoms. Classes D and F were younger (*d*_D_ = -0.43 and *d*_F_ = − 0.41) than other classes, and had lower education. Females were more likely to belong to Class D (62%) than males.

Class E (darkest grey, 11%) was characterized by low baseline self-esteem (4.27) with a neutral trend and high stability (ICC = 0.75). Individuals in this class had marked autonomy-connectedness deficits (Self-awareness *d*_E_ = -1.06, Sensitivity to others *d*_E_ = 0.45, Capacity for managing new situations *d*_E_ = − 0.72) and the highest depressive (*d*_E_ = 0.99) and anxiety (*d*_E_ = 0.88) symptoms. Individuals in Class E were of average age (*d*_E_ = − 0.092), and had lower education than other classes. Females were more likely to belong to Class E (63%) than males.

Sensitivity analyses with time not being restricted in its functional form did not suggest a non-linear relation (see Online Appendix A). Appendix. Fig. 2 in Appendix A displays the results of the sensitivity analysis including all available waves in the LGMM. As can be seen, except for Class C, class compositions are identical. For Class C, there are minor differences with a somewhat higher intercept and smaller slope. We do not judge these differences to be impactful enough to diverge from our initial solution.

As an additional analysis suggested during the review process, we also assessed class differences in socially desirable response tendencies, to explore characteristics of the ceiling class (G). Social desirability was highest in the more ‘confident’ classes (*d*_A_ = 0.27, *d*_B_ = 0.15, *d*_G_ = 0.25, d_F_ = 0.012) and lowest in less ‘confident’ classes (*d*_C_ = − 0.017, *d*_D_ = − 0.28, *d*_E_ = − 0.44), revealing a medium association between self-esteem and providing social desirable responses (*R*^*2*^ = 0.056).

## Discussion

The present study investigated associations between autonomy-connectedness and self-esteem, its developmental trajectories and instability, spanning over 12 years in a large (*N* = 5367), nationally representative population including a wide age range (16 through 91). Results showed that autonomy-connectedness components self-awareness and capacity for managing new situations were moderately and positively associated with self-esteem. Sensitivity to others predicted less deterioration of self-esteem over time. Individual differences were further explored using latent growth mixture modeling and revealed seven latent classes differing in slope and instability over time. Individuals with healthy autonomy were more likely to belong to classes with above average self-esteem, but not necessarily more stable self-esteem trajectories. A class including 11% of the sample population displayed alarming autonomy deficits and low, stable self-esteem, as well as high levels of depressive and anxiety symptoms.

### Autonomy-Connectedness and Self-Esteem Trajectories

In line with the hypotheses, healthy autonomy-connectedness was positively associated with trait self-esteem, as evidenced by correlations between the autonomy-connectedness components and self-esteem, and differences in autonomy-connectedness across classes. Classes characterized by high and stable self-esteem (i.e., A and G) displayed remarkably high self-awareness compared to the other classes, in line with Blatt’s (2008) focus on the importance of self-definition for psychological well-being. The results suggest that a healthy capacity for self-governance seems to correspond with favorable self-esteem trajectories and good mental health. Our findings are also consistent with earlier studies on autonomy-connectedness, showing autonomy deficits in individuals with depression and anxiety symptoms (e.g., Bekker & Belt, 2006; Bekker & Croon, 2010; Kunst et al., 2019; Rutten et al., 2016). The results are less consistent with studies using Beck et al.’s (1983) original ‘autonomy’ subscale (e.g. Bieling et al., 2000), probably due to different operationalizations of autonomy (i.e., ‘self-governance’ in the present study).

The directionality of the association between autonomy-connectedness and self-esteem remains to be elucidated in future research, as we found no clear indications of, for instance, healthy autonomy-connectedness clearly predicting recovering self-esteem trajectories. Thus, both vulnerability and scar mechanisms still seem conceivable based on theory. Individuals with low self-esteem have been described to have global, negative self-beliefs, which can elicit fears and corresponding maladaptive behavioral patterns (e.g., avoidance and pleasing; Fennell, 1997). Low self-esteem could contribute to autonomy deficits when people consistently fail to act upon their authentic needs because of these patterns. Inversely, autonomy deficits could also contribute to lowered self-esteem, because effective self-governance seems important to shape one’s life in a way that benefits (social) need fulfillment, hampering self-esteem (Leary, 2012). Self-neglectful maladaptive behavior can also create and confirm negative self-beliefs (e.g., ‘if my needs are never met, apparently I am not worthwhile’; Fennell, 1997).

High self-esteem on the other hand likely facilitates behaviors corresponding with self-governance (e.g., assertiveness, self-expression; Rutten et al., 2016), which may allow for (social) needs fulfillment and psychological wellbeing, further boosting self-esteem (Leary, 2012). Indications exist that improved interpersonal functioning can, in turn, buffer against even the effects of depression on self-esteem (e.g., Shahar & Davidson, 2003). Studies including multiple autonomy-connectedness and self-esteem measurements may disentangle the interplay between autonomy-connectedness and self-esteem in the development and maintenance of depression and anxiety symptoms.

Partly in line with our hypotheses, sensitivity to others was weakly and positively related to self-esteem in the zero order correlations, and unrelated to self-esteem in the mixed models analyses. For any given individual, being highly sensitive to others’ needs and wishes seems to say little about their self-esteem. At a glance, this finding seems contradictory to earlier studies showing associations between depression and anxiety symptoms on one hand and sociotropy on the other hand (e.g. Bieling et al., 2000; Grondin et al., 2011); as well as anaclitic formulations of psychopathology (Blatt, 2008). However, depression and anxiety symptoms could be most pronounced in individuals with high levels of *maladaptive* others-directedness, whereas the autonomy-connectedness scale seems to measure adaptive as well as maladaptive aspects of connectedness (Kunst et al., 2021). Similarly, earlier studies on interpersonal dependence showed smaller associations between psychopathology and relatively adaptive ‘connectedness’, and larger relations with maladaptive ‘neediness’ (Dunkley et al., 2006).

Related to this, sensitivity to others seemed to ‘buffer’ against the effects of age on declining self-esteem. Across the lifespan, self-esteem gradually decreases from age 50–60 and on (Orth et al., 2018), possibly corresponding with increasing social isolation and employment changes as we age (Orth et al., 2010). Individuals with high sensitivity to others may be more motivated to maintain interpersonal connectedness throughout their lives, thereby keeping a social network to support their sense of relational value (Leary, 2012). All findings on autonomy-connectedness were maintained when controlling for age, education, sex, depressive and anxiety symptoms, and hence are unlikely confounded by these characteristics. However, it should be noted that the buffering effects of sensitivity to others on self-esteem decline were small, and research including measures on life events and quality of social networks seems warranted to gain more insight into these processes.

### Autonomy-Connectedness and Self-Esteem Instability

Whereas mixed models showed that individual differences existed, LGMM allowed for a more detailed exploration and illustration of these differences. The identified classes are in line with earlier research showing weak and negative associations between trait self-esteem and self-esteem instability (Okada, 2010), as classes characterized by high self-esteem were also relatively stable. Additionally, the existence of a ‘low and stable’ class could explain why the association between trait self-esteem and self-esteem instability is merely small, thereby also illustrating the added value of LGMM. Depressive and anxiety symptoms corresponded with the overall self-esteem levels, with most symptoms in the ‘low and stable’ class (E) and less symptoms as classes had higher and more stable self-esteem.

In contrast with our hypotheses, autonomy-connectedness deficits did not necessarily predict *unstable* self-esteem trajectories. Although the most unstable Classes D and F indeed displayed some autonomy deficits (small to medium effects), the most severe autonomy deficits (medium to large effects) were found in the ‘low and stable’ Class E. Our expectation was based on presumed high sensitivity to external evaluations in people with poor capacity for self-governance. However, if such a sensitivity exists, it does not seem visible when assessing trait self-esteem yearly. Momentary fluctuations in state self-esteem were beyond the scope of this study and might be interesting to study in laboratory or experience sampling studies in relation to autonomy-connectedness. Assessed on the long-term, however, severe autonomy deficits seem to correspond with alarmingly low self-esteem scores, high depressive and anxiety symptoms (all large effects), that are unlikely to improve without intervention, possibly due to reciprocal effects of autonomy and self-esteem. Therapies targeting self-esteem (Staring et al., 2016) or autonomy-connectedness (Bekker et al., 2016) might therefore be recommended for this group. An alternative interpretation is that this class is characterized by severe psychopathology or socio-occupational impairments, which collectively contribute to poor self-esteem, autonomy-connectedness and mental health. The directionality of the obtained associations remains to be clarified in future research.

### Comparisons With Previous Latent Class Studies

The present study extended previous LGMM studies by describing not only mental health, but also potential underlying psychological characteristics of self-esteem classes in terms of autonomy-connectedness deficits. We identified more diverse classes based on self-esteem trajectories than in previous studies, possibly due to the nationally representative sample including a wider age range (16–91). In our sample, age was positively associated with self-esteem, and older individuals also showed more decline in self-esteem over time, consistent with meta-analytic research on self-esteem across the lifespan (Orth et al., 2018). The unstable classes (D and F), accordingly, included younger individuals than most other classes. However, it should be noted that we did not find classes *specific* to certain age groups (e.g., adolescents, older individuals), suggesting that the obtained class solutions were not merely reflective of lifespan effects. Nevertheless, our sample contained older individuals than previous studies (ages 12 through 30; Birkeland et al., 2012; Morin et al., 2013; Mund & Neyer, 2016; Oshri et al., 2017; and 16 through 40; Kiviruusu et al., 2016), possibly explaining the identification of a ‘decreasing’ (F) and ‘low and stable’ (E) class. Class G, the ‘ceiling class’, was also a noteworthy variant on the ‘high and stable’ class found previously in LGMM studies. Their high scores did not seem to stem from socially desirable responding tendencies, as social desirability was comparable in other ‘confident’ classes (A and B). This class therefore seems highly content and resilient.

The different latent classes we found compared to earlier studies can also partly be explained by differences in statistical approaches. For instance, we did not obtain the ‘recovering and stabilizing’ classes, that Mund and Neyer (2016) and Morin et al. (2013) did identify. In their studies, non-linear slopes were permitted, thereby allowing trajectories that initially increase and then reach a plateau. Even though our data overall did not support the use of non-linear slopes, Class C (‘high and increasing’) did resemble a ‘stabilizing’ class when non-linear slopes were allowed (see Online Appendix A). Additionally, whereas our, Mund and Neyer (2016) and Morin et al. (2013)’s studies involved error variance in the process of estimating latent classes, other research based the latent classes purely on intercept and slope trajectories (Birkeland et al., 2012; Kiviruusu et al., 2016; Oshri et al., 2017). In these studies, most respondents were classified as ‘high and stable’: 87.4% in Oshri et al. (2017) and 87.1% in Birkeland et al. (2012); compared to 28% in our sample, 29% in Mund and Neyer (2016) and 13.5% in (Morin et al., 2013). Incorporating error variances in class estimation thus allows for the identification of more heterogeneous classes with respect to stability in self-esteem development.

### Clinical Implications

The present study suggests that a relatively large section of the population (11%) suffers from persistent self-esteem and autonomy-connectedness problems. In some of these individuals, self-esteem issues likely preceded the development of their anxiety and depressive symptoms. Prominent social anxiety disorder (SAD) models, for instance, propose that socially anxious individuals have baseline negative self-representations that are activated during conversations, triggering a cascade of cognitive (e.g., overestimation of threat and social cost, self-focused attention) and behavioral (e.g., avoidance, safety behavior) processes that maintain SAD (Hofmann, 2007; Rapee & Heimberg, 1997; Spence & Rapee, 2016). Similarly, low self-esteem tends to precede the development of depressive symptoms (Sowislo & Orth, 2013).

Our findings therefore suggest that negative self-beliefs could be a valuable additional target in psychotherapy for SAD and major depression. Clinicians may evaluate clients' self-esteem histories to determine the potential benefits of adding self-esteem (e.g. Fennell, 1997; Staring et al., 2016) or autonomy-connectedness (Kunst et al., 2022; Maas et al., 2019) enhancing interventions. In cognitive behavioral therapy for SAD, for instance, challenging thoughts about social performance could be accompanied by challenging more global self-beliefs. Cognitive behavioral therapy for SAD typically also involves teaching clients to focus less on ‘what other people think’ during conversations (i.e., decreasing self-focused attention; Spence & Rapee, 2016). Given the large association between self-awareness and self-esteem, a worthwhile venue might be to coach clients with SAD to reflect more on their own interests, needs, preferences and opinions, and to support clients in learning to act accordingly; the main aims of autonomy enhancing treatment (Maas et al., 2019). Developing a stronger sense of self and identity might help clients feel more confident during social interactions and help reduce social anxiety.

### Limitations and Future Directions

The present study was subject to a number of limitations. First, due to the data collection method of the LISS panel the timing of measurement sometimes varied across participants (e.g., T1 was completed either in May or August of 2008). Moreover, four waves had many missing self-esteem measurements and for unclear reasons, and the Rosenberg Self-Esteem Scale used an adapted 7-point scale instead of the original 4-point scale. Second, due to the single autonomy-connectedness measurement, we could not assess changes over time and its associations with self-esteem on timepoints other than 2009. This may be important, as we found that the autonomy-connectedness scores across latent classes were most strongly associated with baseline self-esteem in that class, instead of the increasing or decreasing trend within the class, possibly because of the proximity of the 2009 autonomy-connectedness measure to the first self-esteem measure (in 2008). Theoretically, autonomy-connectedness is viewed as a relatively stable psychological characteristic, stemming from secure attachment experiences (Bekker & van Assen, 2008). Nevertheless, autonomy deficits seem susceptible to positive therapeutic influences (e.g., see Kunst et al., 2022). Its test–retest stability and potential changes across the lifespan remain to be investigated.

Furthermore, we chose to analyze data of all respondents (ages 16–91) without creating subgroups according to life phase, because splitting the sample would reduce the statistical power to detect small to medium meaningful patterns in self-esteem trajectories in the population. We were also unable to use ‘age’ instead of ‘wave’ as a time indicator because of convergence issues. Additionally, our focus was on the role of autonomy-connectedness in self-esteem trajectories, and by incorporating age in the analyses we assessed whether age is associated with self-esteem and its development. Further exploration of the effects of age on self-esteem was beyond the scope of the present study aims. Similarly, we did not take into account potential measurement variance of the self-esteem concept. The RSES factor structure appears largely invariant across nations (e.g., Alessandri et al., 2015; Schmitt & Allik, 2005) as well as across several groups such as clinical versus non-clinical groups (Salerno et al., 2017). Nevertheless, changes in self-esteem could partly reflect differences in interpreting questions and this should be assessed in future research.

Whereas the present study focused on identifying and describing latent classes in terms of autonomy-connectedness, it was beyond of its scope to develop and test models predicting class membership as accurately as possible for specific individuals. Clinically, it would be valuable to predict likely developmental pathways of self-esteem and mental health, based on characteristics that can be targeted in psychotherapy. We regard our study as a first step towards creating optimal prediction models involving more social- and personality variables than those available in our study, to improve the accuracy of the predictions in multinomial regression models. For clinical practice, it would also be interesting to test whether different interventions are required depending on pre-treatment symptom trajectories. We recommend directly comparing effectiveness of autonomy enhancing treatment (Kunst et al., 2022; Maas et al., 2019) to self-esteem boosting therapies (e.g., Staring et al., 2016) to shed light on causal and working mechanisms.

## Conclusion

This study provided insight into individual differences in self-esteem change trajectories and instability, and explored their psychological underpinnings. Individuals who are aware of their needs and wishes and who can manage new situations well, also seem to have higher self-esteem. Whereas previous literature on autonomy showed that being ‘dependent’ on others may represent vulnerability for depression and anxiety, our results suggest that ‘caring what people think’ (i.e., being highly sensitive to others’ needs, wishes and opinions) also seems adaptive in terms of buffering against declining self-esteem in older age. Unexpectedly, severe autonomy deficits were not necessarily related to *unstable* trait self-esteem, but mostly to low and stable self-esteem. Individuals with poor capacity for self-governance perhaps show great difficulty designing their lives in accordance to their (inter)personal needs and may therefore struggle to improve their self-esteem and mental health. Interventions targeting autonomy and self-esteem may be promising in these groups (e.g., Kunst et al., 2022; Maas et al., 2019; Staring et al., 2016).

## Supplementary Information

Below is the link to the electronic supplementary material.

Supplementary Material 1.

## Data Availability

Utilized data and syntax files are available to any researcher wishing to use them for non-commercial purposes. The data files are property of CentERdata (Tilburg University, the Netherlands) and are available through www.lissdata.nl. The utilized syntax files are available through 10.34894/4NDGHW and upon request from the corresponding author.
