# Supplementary material for: Who Cares What Other People Think? A Longitudinal Investigation of the Role of Autonomy-Connectedness in Self-Esteem Change Trajectories and Instability
Source: Cognit Ther Res. 2025 Apr 28;49(6):1246–63. doi: 10.1007/s10608-025-10604-2 (PMC12638343; doi:10.1007/s10608-025-10604-2)

# Appendix A. Online Supplementary Material

**For: *Who* Cares What Other People Think? A Longitudinal Investigation of the Role of Autonomy-Connectedness in Self-Esteem Change Trajectories and Instability**

**Sensitivity Analyses Mixed Models**

Three sensitivity analyses were conducted, all with Model 4 (see main manuscript) as starting point and increasing data or model complexity:

1. Model 4 was estimated on all data, using data of all 12 timepoints (waves) instead of 8. See Appendix.Table 1.
2. For Model 5, Model 4 was repeated, also controlling for background variables: effects of age, sex, net household income and education (categorical), which were added to Model 4 (using the original 8 timepoints) as fixed covariates (adding 8 parameters). See Appendix.Table 2.
3. In Model 6, in addition to the other control variables, we also adding (centered) depressive and anxiety symptoms and their interactions with time to Model 4 (also using the original 8 timepoints), to verify whether developments in self-esteem were also predicted by depressive and anxiety symptoms, and whether the results on autonomy-connectedness were maintained. See Appendix.Table 2.

As a final addition, for interpretation purposes, we examined the effects of Age on self-esteem development (page 3, Appendix). Sensitivity analyses including all timepoints did not alter the results (Appendix.Table 1). Adding demographic characteristics age, sex, net income of the household and education level to Model 4 improved model fit (*-2LL* = 48,502.95, *χ²*(8) = 4,156.72, *p* < .001; *BIC* = 48,766.30), but did not alter the results on autonomy-connectedness (Model 5, Appendix.Table 2). Age was positively associated with self-esteem (*B* = 0.0045, *SE* = .00064, *p* < .001), but older individuals also showed more decline in self-esteem over time (time x age interaction: *B* = -0.0041, *SE* = 0.00092, *p* < .001). Having completed primary education only was associated with lower self-esteem when compared to having a university degree (*B* = -.23, *SE* = .051, *p* < .001; Model 5, Appendix.Table 2).

**Appendix.Table 1**

|  | Model 4 (8 waves)  *N* = 5171 | | | Model 4 (12 waves)  *N* = 5367 | | |
| --- | --- | --- | --- | --- | --- | --- |
| *Fixed effects* | *B* | *SE B* | *p* | *B* | *SE B* | *p* |
| Intercept | 5.62 | 0.11 | < .001 | 5.62 | 0.011 | < .001 |
| Time | -0.080 | 0.014 | < .001 | -0.080 | 0.014 | < .001 |
| Self-awareness | 0.53 | 0.017 | < .001 | 0.52 | 0.016 | < .001 |
| Sensitivity to others | -0.092 | 0.022 | < .001 | -0.096 | 0.022 | < .001 |
| Capacity for managing new situations | 0.19 | 0.014 | < .001 | 0.20 | 0.014 | < .001 |
| Sensitivity to others×Time | 0.10 | 0.026 | < .001 | 0.11 | 0.025 | < .001 |
|  |  |  |  |  |  |  |
| *Random effects* |  | | |  | | |
| Intercept variance | 0.42 | | | 0.43 | | |
| Slope (time) variance | 0.28 | | | 0.28 | | |
| Intercept-time covariance | -0.039 | | | -0.038 | | |
|  |  | | |  | | |
| *Model fit indices* |  | | |  | | |
|  | *-2LL* = 52,659.67  *χ²*(1) *=* 16.37, *p* < .001  *BIC* = 52,843.36 | | | *-2LL* = 55,358.85  *χ²*(1) *=* 18.74, *p* < .001  *BIC* = 55,584.47 | | |
|  |  |  |  |  |  |  |
| Number of parameters | 18 | | | 22 | | |
|  |  | | |  | | |

*Sensitivity Analyses on the Predictive Effects of Time and Autonomy-Connectedness on Self-Esteem for 8 and 12 Waves*

*Note.* Unit of time = per 10 years. The -2LL for Model 4 (8 waves) and Model 4 (12 waves) were compared to versions of the model excluding the Sensitivity to others × Time interaction term (Model 2, main manuscript).

Depressive and anxiety symptoms (measured in 2009) were added to Model 4 (Model 6, Appendix.Table 2). This model again showed improved fit (*-2LL* = 47,868.18, *χ²*(2) = 634.77, *p* < .001; *BIC* = 48,151.78), with both depressive (*B* = -0.039, *SE* = 0.0023, *p* < .001) and anxiety (*B* = -0.016, *SE* = 0.0025, *p* < .001) symptoms being negatively associated with self-esteem. Main results on effects of autonomy-connectedness on Self-esteem were unaffected, that is: Self-awareness (*B* = 0.42, *SE* = 0.016, *p* < .001) and Capacity for managing new situations (*B* = 0.13, *SE* = 0.014, *p* < .001) still predicted self-esteem, and there was a positive interaction effect between Sensitivity to others and time (*B* = 0.10, *SE* = 0.027, *p* < .001). No interactions between depressive symptoms and time, and anxiety symptoms and time were found (Model 7, Appendix.Table 2), suggesting that the significant time x sensitivity to others interaction was not confounded by effects of depressive or anxiety symptoms on self-esteem development over time.

|  | Model 5  *N* = 4791 | | | | Model 6  *N* = 4791 | | | Model 7  *N* = 4791 | | |
| --- | --- | --- | --- | --- | --- | --- | --- | --- | --- | --- |
| *Fixed effects* | *B* | | *SE B* | *p* | *B* | *SE B* | *p* | *B* | *SE B* | *p* |
| Intercept | 5.44 | | 0.050 | < .001 | 5.37 | 0.048 | < .001 | 5.37 | 0.048 | < .001 |
| Time | -0.084 | | 0.014 | < .001 | -0.083 | 0.014 | < .001 | -0.082 | 0.014 | < .001 |
| Self-awareness | 0.51 | | 0.017 | < .001 | 0.42 | 0.016 | < .001 | 0.42 | 0.016 | < .001 |
| Sensitivity to others | -0.12 | | 0.024 | < .001 | -0.042 | 0.023 | .064 | -0.036 | 0.023 | .111 |
| CMNS | 0.19 | | 0.015 | < .001 | 0.13 | 0.014 | < .001 | 0.13 | 0.014 | < .001 |
| Sensitivity to others×Time | 0.10 | | 0.027 | < .001 | 0.10 | 0.027 | < .001 | 0.086 | 0.028 | .002 |
|  |  | |  |  |  |  |  |  |  |  |
| Age | 0.0045 | | 0.00063 | < .001 | 0.0054 | 0.00061 | < .001 | 0.0054 | 0.00061 | < .001 |
| Sex | 0.045 | | 0.022 | 0.043 | 0.061 | 0.021 | .003 | 0.062 | 0.021 | .003 |
| Education |  | |  |  |  |  |  |  |  |  |
| Primary | -0.23 | | 0.051 | < .001 | -0.17 | 0.047 | < .001 | -0.17 | 0.047 | < .001 |
| Intermediate sec. | -0.11 | | 0.043 | 0.0076 | -0.086 | 0.040 | .033 | -0.086 | 0.040 | .032 |
| Higher sec. | -0.13 | | 0.049 | 0.0091 | -0.12 | 0.046 | .012 | -0.12 | 0.046 | .012 |
| Intermediate voc. | -0.053 | | 0.044 | 0.23 | -0.029 | 0.041 | .474 | -0.029 | 0.041 | .473 |
| Higher voc. | 0.074 | | 0.043 | 0.086 | 0.064 | 0.040 | .116 | 0.063 | 0.040 | .118 |
| Household income | 2E-06 | | 2E-06 | .282 | 5.72E-07 | 2E-06 | .715 | 5.6822E-7 | 2E-06 | .716 |
|  |  | |  |  |  |  |  |  |  |  |
| Anxiety symp |  | |  |  | -0.016 | 0.0025 | < .001 | -0.017 | 0.0027 | < .001 |
| Depression symp |  | |  |  | -0.039 | 0.0023 | < .001 | -0.040 | 0.0025 | < .001 |
| AnxietyxTime |  | |  |  |  |  |  | 0.0043 | 0.0036 | .243 |
| DepressionxTime |  | |  |  |  |  |  | 0.0027 | 0.0033 | .406 |
|  |  | |  |  |  |  |  |  |  |  |
| *Random effects* | |  | | |  | | |  |  |  |
| Intercept variance | | 0.41 | | | 0.34 | | | 0.34 | | |
| Slope (time) variance | | 0.28 | | | 0.28 | | | 0.28 | | |
| Intercept-time covariance | | -0.037 | | | -0.032 | | | -0.032 | | |
|  |  | | | |  | | |  | | |
| *Model fit indices* | |  | | |  | | |  | | |
|  | *-2LL* = 48,502.95  *χ²*(8) = 4156.72, *p* < .001  *BIC* = 48,766.30 | | | | *-2LL* = 47,868.18  *χ²*(2) = 634.77, *p* < .001  *BIC* = 48,151.78 | | | *-2LL* = 47,863.030  *χ²*(2) = 5.15, *p* = .0762  *BIC* = 48,166.89 | | |
|  |  |  |  |  |  |  |  |  |  |  |
| No. of parameters | 26 | | | | 28 | | | 30 | | |

**Appendix.Table 2**

*Sensitivity Analyses on the Predictive Effects of Time and Autonomy-Connectedness on Self-Esteem*

*Note:* Model 5 was compared to Model 4 (8 waves; Appendix.Table 1). Reference category for education is university degree. CMNS = Capacity for managing new situations; sec. = secondary; voc. = vocational.

N.B.: adding an Age x Time interaction to Model 6 revealed a significant negative Age x Time interaction, *B* = -0.0041, *SE* = 0.00092, *t*(3563.69) = -4.45, *p* < .001, so that older individuals showed more pronounced decreases in self-esteem over time (*-2LL* = 47,848.41, *χ²*(1) = 19.77, *p* < .001 , *BIC* = 48,142.14).

**Sensitivity Analyses Latent Growth Mixture Modelling (LGMM)**

Sensitivity analyses with time not being restricted in its functional form were performed, but did not suggest a non-linear relation.

**Appendix.Table 4**

*2-Class Solution With Linear Effect of Time*

|  | Class1 | Class2 | Wald | p-value |
| --- | --- | --- | --- | --- |
| Intercept |  |  |  |  |
| Mean | 5.1657 | 6.2573 | 337902.2030 | < 0.001 |
| Random | 0.6883 | 0.3875 | 7955.6688 | < 0.001 |
| Slope | -0.0203 | -0.0031 | 57.8165 | < 0.001 |
| Error Variance | 0.4521 | 0.0986 |  |  |

**Appendix.Table 3**

*1-Class Solution With Linear Effect of Time*

|  | Class1 | Wald | p-value |
| --- | --- | --- | --- |
| Intercept |  |  |  |
| Mean | 5.6390 | 220065.4258 | < 0.001 |
| Random | 0.7368 | 15809.9850 | < 0.001 |
| Slope | -0.0126 | 57.2787 | < 0.001 |
| Error Variance | 0.3038 |  |  |

**Appendix.Table 5**

*3-Class Solution With Linear Effect of Time*

|  | Class1 | Class2 | Class3 | Wald | p-value |
| --- | --- | --- | --- | --- | --- |
| Intercept |  |  |  |  |  |
| Mean | 5.0950 | 6.1162 | 6.8221 | 142445.3897 | < 0.001 |
| Random | 0.6746 | 0.3589 | 0.1127 | 4918.1836 | < 0.001 |
| Slope | -0.0230 | -0.0025 | -0.0019 | 58.9391 | < 0.001 |
| Error Variance | 0.4762 | 0.1252 | 0.0213 |  |  |

**Appendix.Table 6**

*4-Class Solution With Linear Effect of Time*

|  | Class1 | Class2 | Class3 | Class4 | Wald | p-value |
| --- | --- | --- | --- | --- | --- | --- |
| Intercept |  |  |  |  |  |  |
| Mean | 5.2058 | 6.2549 | 5.2416 | 6.8611 | 382800.8703 | < 0.001 |
| Random | 0.6696 | 0.3118 | 0.6753 | 0.0845 | 4788.2287 | < 0.001 |
| Slope | 0.0020 | -0.0063 | -0.0596 | -0.0014 | 52.9514 | < 0.001 |
| Error Variance | 0.2613 | 0.1036 | 0.8009 | 0.0170 |  |  |

**Appendix.Table 7**

*5-Class Solution With Linear Effect of Time*

|  | Class1 | Class2 | Class3 | Class4 | Class5 | Wald | p-value |
| --- | --- | --- | --- | --- | --- | --- | --- |
| Intercept |  |  |  |  |  |  |  |
| Mean | 6.2299 | 5.2934 | 4.7597 | 5.5868 | 6.8598 | 424845.4239 | < 0.001 |
| Random | 0.3235 | 0.6948 | 0.6696 | 0.5781 | 0.0859 | 7392.7178 | < 0.001 |
| Slope | -0.0049 | -0.0660 | 0.1316 | -0.1362 | -0.0016 | 913.8394 | < 0.001 |
| Error Variance | 0.1065 | 0.2411 | 0.3225 | 0.8799 | 0.0171 |  |  |

**Appendix.Table 8**

*6-Class Solution With Linear Effect of Time*

|  | Class1 | Class2 | Class3 | Class4 | Class5 | Class6 | Wald | p-value |
| --- | --- | --- | --- | --- | --- | --- | --- | --- |
| Intercept |  |  |  |  |  |  |  |  |
| Mean | 6.2492 | 5.8816 | 4.8118 | 5.4353 | 4.3018 | 6.8685 | 404010.1892 | < 0.001 |
| Random | 0.3116 | 0.4223 | 0.5717 | 0.6477 | 0.4954 | 0.0825 | 2942.4353 | < 0.001 |
| Slope | 0.0011 | -0.0684 | 0.1404 | -0.1447 | -0.0056 | -0.0018 | 841.7740 | < 0.001 |
| Error Variance | 0.0973 | 0.2252 | 0.3529 | 0.7661 | 0.1535 | 0.0158 |  |  |

**Appendix.Table 9**

*7-Class Solution With Linear Effect of Time*

|  | Class1 | Class2 | Class3 | Class4 | Class5 | Class6 | Class7 | Wald | p-value |
| --- | --- | --- | --- | --- | --- | --- | --- | --- | --- |
| Intercept |  |  |  |  |  |  |  |  |  |
| Mean | 6.3709 | 5.9258 | 5.5135 | 4.6338 | 4.2722 | 5.6404 | 6.8720 | 331426.1073 | < 0.001 |
| Random | 0.2815 | 0.4197 | 0.4203 | 0.5259 | 0.4857 | 0.6504 | 0.0753 | 2649.3019 | < 0.001 |
| Slope | -0.0155 | -0.0718 | 0.0852 | 0.1346 | 0.0003 | -0.2014 | -0.0002 | 913.3826 | < 0.001 |
| Error Variance | 0.0843 | 0.2227 | 0.1572 | 0.6084 | 0.1619 | 0.6213 | 0.0149 |  |  |

**Appendix.Table 10**

*8-Class Solution With Linear Effect of Time*

|  | Class1 | Class2 | Class3 | Class4 | Class5 | Class6 | Class7 | Class8 | Wald | p-value |
| --- | --- | --- | --- | --- | --- | --- | --- | --- | --- | --- |
| Intercept |  |  |  |  |  |  |  |  |  |  |
| Mean | 5.8405 | 6.3759 | 5.6654 | 4.6351 | 4.2611 | 5.6152 | 6.7976 | 6.9727 | 614052.9593 | < 0.001 |
| Random | 0.4257 | 0.2706 | 0.3846 | 0.5266 | 0.4866 | 0.6697 | 0.0709 | 0.0168 | 2766.9549 | < 0.001 |
| Slope | -0.0669 | -0.0249 | 0.0811 | 0.1409 | 0.0032 | -0.2000 | 0.0012 | 0.0000 | 1068.1524 | < 0.001 |
| Error Variance | 0.2352 | 0.0882 | 0.1362 | 0.5666 | 0.1583 | 0.6441 | 0.0269 | 0.0028 |  |  |

**Appendix.Table 11**

*9-Class Solution With Linear Effect of Time*

|  | Class1 | Class2 | Class3 | Class4 | Class5 | Class6 | Class7 | Class8 | Class9 | Wald | p-value |
| --- | --- | --- | --- | --- | --- | --- | --- | --- | --- | --- | --- |
| Intercept |  |  |  |  |  |  |  |  |  |  |  |
| Mean | 5.8926 | 6.3932 | 4.7643 | 5.7465 | 4.2719 | 5.7151 | 6.8062 | 4.6632 | 6.9761 | 494846.9826 | < 0.001 |
| Random | 0.4036 | 0.2653 | 0.5031 | 0.3731 | 0.4907 | 0.5898 | 0.0694 | 0.8185 | 0.0146 | 2296.2578 | < 0.001 |
| Slope | -0.0587 | -0.0260 | 0.1283 | 0.0793 | 0.0007 | -0.2042 | 0.0007 | 0.0037 | -0.0001 | 960.3277 | < 0.001 |
| Error Variance | 0.2175 | 0.0856 | 0.4560 | 0.1193 | 0.1595 | 0.5065 | 0.0256 | 1.4741 | 0.0025 |  |  |

**Appendix.Table 12**

*10-Class Solution With Linear Effect of Time*

|  | Class1 | Class2 | Class3 | Class4 | Class5 | Class6 | Class7 | Class8 | Class9 | Class10 | Wald | p-value |
| --- | --- | --- | --- | --- | --- | --- | --- | --- | --- | --- | --- | --- |
| Intercept |  |  |  |  |  |  |  |  |  |  |  |  |
| Mean | 5.6683 | 6.3223 | 5.5676 | 4.7132 | 5.7658 | 6.5880 | 3.9577 | 4.1992 | 6.8740 | 6.9929 | 969406.6695 | < 0.001 |
| Random | 0.5173 | 0.2564 | 0.3961 | 0.4214 | 0.6341 | 0.0983 | 0.6320 | 0.2652 | 0.0190 | 0.0000 | 2060.4341 | < 0.001 |
| Slope | -0.0818 | -0.0369 | 0.0725 | 0.1365 | -0.2287 | 0.0094 | 0.0363 | 0.0016 | 0.0006 | -0.0011 | 991.5428 | < 0.001 |
| Error Variance | 0.2560 | 0.0991 | 0.1594 | 0.6405 | 0.7029 | 0.0613 | 0.2650 | 0.0773 | 0.0176 | 0.0015 |  |  |

**Appendix.Table 14**

*2-Class Solution With Unrestricted Effect of Time*

|  | Class1 | Class2 | Wald | p-value |
| --- | --- | --- | --- | --- |
| Intercept |  |  |  |  |
| Mean | 5.1656 | 6.2714 | 305049.5352 | < 0.001 |
| Random | 0.6885 | 0.3878 | 8024.4143 | < 0.001 |
| Slope |  |  |  |  |
| 1 | 0 | 0 | 110.9687 | < 0.001 |
| 2 | -0.0051 | -0.0261 |  |  |
| 3 | -0.1237 | -0.0516 |  |  |
| 4 | -0.0801 | -0.0238 |  |  |
| 5 | -0.1039 | -0.0127 |  |  |
| 6 | -0.1195 | -0.0345 |  |  |
| 7 | -0.1530 | -0.0301 |  |  |
| 8 | -0.1271 | -0.0431 |  |  |
| Error Variance | 0.4511 | 0.0983 |  |  |

**Appendix.Table 13**

*1-Class Solution With Unrestricted Effect of Time*

|  | Class1 | Wald | p-value |
| --- | --- | --- | --- |
| Intercept |  |  |  |
| Mean | 5.6463 | 188863.2193 | < 0.001 |
| Random | 0.7365 | 15787.4027 | < 0.001 |
| Slope |  |  |  |
| 1 | 0 | 99.9457 | < 0.001 |
| 2 | -0.0148 |  |  |
| 3 | -0.0932 |  |  |
| 4 | -0.0560 |  |  |
| 5 | -0.0644 |  |  |
| 6 | -0.0848 |  |  |
| 7 | -0.0975 |  |  |
| 8 | -0.0900 |  |  |
| Error Variance | 0.3033 |  |  |

**Appendix.Table 15**

*3-Class Solution With Unrestricted Effect of Time*

|  | Class1 | Class2 | Class3 | Wald | p-value |
| --- | --- | --- | --- | --- | --- |
| Intercept |  |  |  |  |  |
| Mean | 5.0929 | 6.1341 | 6.8220 | 128866.2198 | < 0.001 |
| Random | 0.6750 | 0.3587 | 0.1122 | 4976.8484 | < 0.001 |
| Slope |  |  |  |  |  |
| 1 | 0 | 0 | 0 | 115.4739 | < 0.001 |
| 2 | -0.0060 | -0.0252 | -0.0052 |  |  |
| 3 | -0.1293 | -0.0591 | -0.0103 |  |  |
| 4 | -0.0898 | -0.0214 | 0.0000 |  |  |
| 5 | -0.1170 | -0.0085 | -0.0126 |  |  |
| 6 | -0.1340 | -0.0344 | 0.0085 |  |  |
| 7 | -0.1704 | -0.0292 | -0.0078 |  |  |
| 8 | -0.1409 | -0.0401 | -0.0287 |  |  |
| Error Variance | 0.4747 | 0.1247 | 0.0211 |  |  |

**Appendix.Table 16**

*4-Class Solution With Unrestricted Effect of Time*

|  | Class1 | Class2 | Class3 | Class4 | Wald | p-value |
| --- | --- | --- | --- | --- | --- | --- |
| Intercept |  |  |  |  |  |  |
| Mean | 5.2384 | 6.2688 | 5.1948 | 6.8678 | 333558.9458 | < 0.001 |
| Random | 0.6669 | 0.3129 | 0.6861 | 0.0837 | 4840.2965 | < 0.001 |
| Slope |  |  |  |  |  |  |
| 1 | 0 | 0 | 0 | 0 | 116.6769 | < 0.001 |
| 2 | -0.0178 | -0.0274 | 0.0160 | -0.0109 |  |  |
| 3 | -0.0724 | -0.0559 | -0.2146 | -0.0187 |  |  |
| 4 | -0.0025 | -0.0329 | -0.2192 | -0.0045 |  |  |
| 5 | 0.0191 | -0.0273 | -0.3178 | -0.0219 |  |  |
| 6 | 0.0204 | -0.0581 | -0.3663 | 0.0046 |  |  |
| 7 | -0.0007 | -0.0542 | -0.4072 | -0.0031 |  |  |
| 8 | 0.0100 | -0.0677 | -0.3582 | -0.0293 |  |  |
| Error Variance | 0.2585 | 0.1023 | 0.7794 | 0.0166 |  |  |

**Appendix.Table 17**

*5-Class Solution With Unrestricted Effect of Time*

|  | Class1 | Class2 | Class3 | Class4 | Class5 | Wald | p-value |
| --- | --- | --- | --- | --- | --- | --- | --- |
| Intercept |  |  |  |  |  |  |  |
| Mean | 6.2420 | 5.2565 | 4.8312 | 5.5238 | 6.8653 | 341941.6865 | < 0.001 |
| Random | 0.3243 | 0.6912 | 0.6684 | 0.5842 | 0.0855 | 7435.7934 | < 0.001 |
| Slope |  |  |  |  |  |  |  |
| 1 | 0 | 0 | 0 | 0 | 0 | 1088.3067 | < 0.001 |
| 2 | -0.0269 | -0.0620 | 0.1445 | -0.0789 | -0.0105 |  |  |
| 3 | -0.0530 | -0.2276 | 0.2753 | -0.4251 | -0.0181 |  |  |
| 4 | -0.0287 | -0.2607 | 0.5690 | -0.5347 | -0.0046 |  |  |
| 5 | -0.0191 | -0.2852 | 0.6771 | -0.7328 | -0.0215 |  |  |
| 6 | -0.0484 | -0.3369 | 0.7857 | -0.8452 | 0.0042 |  |  |
| 7 | -0.0409 | -0.4398 | 0.8782 | -0.8608 | -0.0041 |  |  |
| 8 | -0.0521 | -0.4266 | 0.8867 | -0.8015 | -0.0308 |  |  |
| Error Variance | 0.1063 | 0.2387 | 0.3168 | 0.8411 | 0.0168 |  |  |

**Appendix.Table 18**

*6-Class Solution With Unrestricted Effect of Time*

|  | Class1 | Class2 | Class3 | Class4 | Class5 | Class6 | Wald | p-value |
| --- | --- | --- | --- | --- | --- | --- | --- | --- |
| Intercept |  |  |  |  |  |  |  |  |
| Mean | 6.2694 | 5.8464 | 4.8759 | 5.3721 | 4.3273 | 6.8777 | 328052.9334 | < 0.001 |
| Random | 0.3091 | 0.4207 | 0.5844 | 0.6373 | 0.4967 | 0.0817 | 2974.4714 | < 0.001 |
| Slope |  |  |  |  |  |  |  |  |
| 1 | 0 | 0 | 0 | 0 | 0 | 0 | 1022.786 | < 0.001 |
| 2 | -0.0210 | -0.0708 | 0.1647 | -0.0786 | -0.0131 | -0.0135 |  |  |
| 3 | -0.0262 | -0.2215 | 0.2951 | -0.4388 | -0.0711 | -0.0212 |  |  |
| 4 | 0.0005 | -0.2228 | 0.6142 | -0.5862 | -0.0636 | -0.0085 |  |  |
| 5 | 0.0118 | -0.2443 | 0.7263 | -0.7485 | -0.0732 | -0.0291 |  |  |
| 6 | -0.0113 | -0.3324 | 0.8537 | -0.8520 | -0.0358 | 0.0000 |  |  |
| 7 | 0.0069 | -0.4485 | 0.9561 | -0.8837 | -0.0254 | -0.0057 |  |  |
| 8 | -0.0109 | -0.4214 | 0.9640 | -0.8246 | -0.0676 | -0.0320 |  |  |
| Error Variance | 0.0951 | 0.2193 | 0.3394 | 0.7288 | 0.1531 | 0.0151 |  |  |

**Appendix.Table 19**

*7-Class Solution With Unrestricted Effect of Time*

|  | Class1 | Class2 | Class3 | Class4 | Class5 | Class6 | Class7 | Wald | p-value |
| --- | --- | --- | --- | --- | --- | --- | --- | --- | --- |
| Intercept |  |  |  |  |  |  |  |  |  |
| Mean | 5.8281 | 6.3864 | 5.5149 | 4.7039 | 4.2874 | 5.6659 | 6.8797 | 288501.5317 | < 0.001 |
| Random | 0.4221 | 0.2884 | 0.6472 | 0.5407 | 0.4885 | 0.3847 | 0.0762 | 2948.0507 | < 0.001 |
| Slope |  |  |  |  |  |  |  |  |  |
| 1 | 0 | 0 | 0 | 0 | 0 | 0 | 0 | 1386.3914 | < 0.001 |
| 2 | -0.0804 | -0.0463 | -0.1428 | 0.2015 | -0.0012 | 0.1441 | -0.0113 |  |  |
| 3 | -0.2113 | -0.0930 | -0.5868 | 0.2894 | -0.0396 | 0.3092 | -0.0173 |  |  |
| 4 | -0.2080 | -0.1049 | -0.7698 | 0.5702 | -0.0142 | 0.5407 | -0.0008 |  |  |
| 5 | -0.2118 | -0.1026 | -0.9659 | 0.6896 | -0.0240 | 0.5603 | -0.0235 |  |  |
| 6 | -0.2955 | -0.1369 | -1.1121 | 0.8294 | 0.0202 | 0.6231 | 0.0088 |  |  |
| 7 | -0.3862 | -0.1324 | -1.2217 | 1.0084 | 0.0304 | 0.6567 | 0.0019 |  |  |
| 8 | -0.3562 | -0.1546 | -1.1458 | 1.0120 | -0.0118 | 0.6427 | -0.0186 |  |  |
| Error Variance | 0.2253 | 0.0827 | 0.6145 | 0.5416 | 0.1572 | 0.1127 | 0.0145 |  |  |

**Appendix.Table 20**

*8-Class Solution With Unrestricted Effect of Time*

|  | Class1 | Class2 | Class3 | Class4 | Class5 | Class6 | Class7 | Class8 | Wald | p-value |
| --- | --- | --- | --- | --- | --- | --- | --- | --- | --- | --- |
| Intercept |  |  |  |  |  |  |  |  |  |  |
| Mean | 5.8021 | 6.3726 | 5.5131 | 4.7009 | 4.2817 | 5.6638 | 6.8010 | 6.9683 | 541852.4745 | < 0.001 |
| Random | 0.4192 | 0.2728 | 0.6516 | 0.5429 | 0.4865 | 0.3810 | 0.0707 | 0.0171 | 2920.2857 | < 0.001 |
| Slope |  |  |  |  |  |  |  |  |  |  |
| 1 | 0 | 0 | 0 | 0 | 0 | 0 | 0 | 0 | 1410.4392 | < 0.001 |
| 2 | -0.0807 | -0.0520 | -0.1443 | 0.2044 | -0.0007 | 0.1363 | -0.0011 | 0.0162 |  |  |
| 3 | -0.2129 | -0.1048 | -0.5912 | 0.2912 | -0.0378 | 0.3018 | -0.0054 | -0.0053 |  |  |
| 4 | -0.2105 | -0.1175 | -0.7750 | 0.5732 | -0.0122 | 0.5288 | 0.0144 | 0.0070 |  |  |
| 5 | -0.2140 | -0.1167 | -0.9736 | 0.6924 | -0.0224 | 0.5496 | -0.0024 | 0.0134 |  |  |
| 6 | -0.2987 | -0.1576 | -1.1186 | 0.8337 | 0.0232 | 0.6113 | 0.0320 | 0.0027 |  |  |
| 7 | -0.3942 | -0.1505 | -1.2257 | 1.0133 | 0.0339 | 0.6459 | 0.0076 | 0.0069 |  |  |
| 8 | -0.3635 | -0.1723 | -1.1494 | 1.0169 | -0.0083 | 0.6321 | -0.0125 | -0.0001 |  |  |
| Error Variance | 0.2290 | 0.0881 | 0.6179 | 0.5430 | 0.1564 | 0.1138 | 0.0262 | 0.0026 |  |  |

**Appendix.Table 21**

*9-Class Solution With Unrestricted Effect of Time*

|  | Class1 | Class2 | Class3 | Class4 | Class5 | Class6 | Class7 | Class8 | Class9 | Wald | p-value |
| --- | --- | --- | --- | --- | --- | --- | --- | --- | --- | --- | --- |
| Intercept |  |  |  |  |  |  |  |  |  |  |  |
| Mean | 5.7860 | 6.3900 | 4.5975 | 5.2677 | 5.7842 | 4.3212 | 5.3862 | 6.8102 | 6.9752 | 635914.9360 | < 0.001 |
| Random | 0.4152 | 0.2735 | 0.6844 | 0.7376 | 0.3454 | 0.3929 | 0.2718 | 0.0706 | 0.0113 | 1935.9309 | < 0.001 |
| Slope |  |  |  |  |  |  |  |  |  |  |  |
| 1 | 0 | 0 | 0 | 0 | 0 | 0 | 0 | 0 | 0 | 1091.5208 | < 0.001 |
| 2 | -0.0720 | -0.0549 | 0.1795 | -0.1096 | 0.1234 | -0.0069 | 0.0154 | -0.0026 | 0.0151 |  |  |
| 3 | -0.1802 | -0.1175 | 0.2561 | -0.5959 | 0.2951 | -0.0182 | -0.1124 | -0.0058 | -0.0074 |  |  |
| 4 | -0.1708 | -0.1352 | 0.6124 | -0.8272 | 0.4984 | 0.0134 | -0.1090 | 0.0129 | 0.0071 |  |  |
| 5 | -0.1783 | -0.1358 | 0.7735 | -0.9293 | 0.5215 | -0.0032 | -0.3349 | -0.0032 | 0.0129 |  |  |
| 6 | -0.2485 | -0.1759 | 0.9345 | -1.1128 | 0.5657 | 0.0350 | -0.3223 | 0.0276 | 0.0053 |  |  |
| 7 | -0.3276 | -0.1730 | 1.0707 | -1.2638 | 0.6069 | 0.0303 | -0.1927 | 0.0066 | 0.0052 |  |  |
| 8 | -0.2951 | -0.1956 | 1.0904 | -1.2299 | 0.5845 | -0.0245 | -0.1231 | -0.0143 | -0.0031 |  |  |
| Error Variance | 0.2309 | 0.0857 | 0.3824 | 0.4363 | 0.1007 | 0.1319 | 1.0441 | 0.0245 | 0.0021 |  |  |

**Appendix.Table 22**

*10-Class Solution With Unrestricted Effect of Time*

|  | Class1 | Class2 | Class3 | Class4 | Class5 | Class6 | Class7 | Class8 | Class9 | Class10 | Wald | p-value |
| --- | --- | --- | --- | --- | --- | --- | --- | --- | --- | --- | --- | --- |
| Intercept |  |  |  |  |  |  |  |  |  |  |  |  |
| Mean | 5.7957 | 6.3944 | 4.6539 | 5.8147 | 5.6163 | 4.3059 | 5.2783 | 4.4620 | 6.8129 | 6.9781 | 729815.1901 | < 0.001 |
| Random | 0.4180 | 0.2739 | 0.6283 | 0.3375 | 0.5360 | 0.3702 | 0.3140 | 0.7086 | 0.0706 | 0.0110 | 2325.2722 | < 0.001 |
| Slope |  |  |  |  |  |  |  |  |  |  |  |  |
| 1 | 0 | 0 | 0 | 0 | 0 | 0 | 0 | 0 | 0 | 0 | 1219.4863 | < 0.001 |
| 2 | -0.0718 | -0.0547 | 0.1980 | 0.1143 | -0.0907 | -0.0242 | 0.0017 | -0.0373 | -0.0028 | 0.0138 |  |  |
| 3 | -0.1701 | -0.1202 | 0.2767 | 0.2866 | -0.6565 | 0.0072 | -0.0463 | -0.3159 | -0.0056 | -0.0097 |  |  |
| 4 | -0.1611 | -0.1395 | 0.6776 | 0.4809 | -0.8472 | 0.0815 | -0.0445 | -0.5132 | 0.0130 | 0.0053 |  |  |
| 5 | -0.1695 | -0.1405 | 0.8406 | 0.5047 | -0.9821 | 0.0594 | -0.2362 | -0.5278 | -0.0031 | 0.0113 |  |  |
| 6 | -0.2431 | -0.1796 | 0.9830 | 0.5457 | -1.3235 | 0.0741 | -0.1492 | -0.3574 | 0.0266 | 0.0055 |  |  |
| 7 | -0.3238 | -0.1766 | 1.1004 | 0.5884 | -1.5680 | 0.0347 | 0.0198 | -0.2539 | 0.0065 | 0.0047 |  |  |
| 8 | -0.2909 | -0.2000 | 1.1114 | 0.5650 | -1.5452 | -0.0112 | 0.0932 | -0.2431 | -0.0144 | -0.0051 |  |  |
| Error Variance | 0.2292 | 0.0847 | 0.3520 | 0.0979 | 0.4347 | 0.1102 | 1.0454 | 0.3001 | 0.0240 | 0.0019 |  |  |

**Appendix.Figure 1**

*Spaghetti Plots of Sensitivity Analysis Using Dummy Time Variables (i.e., Not Restricting Time to a Linear Form)*


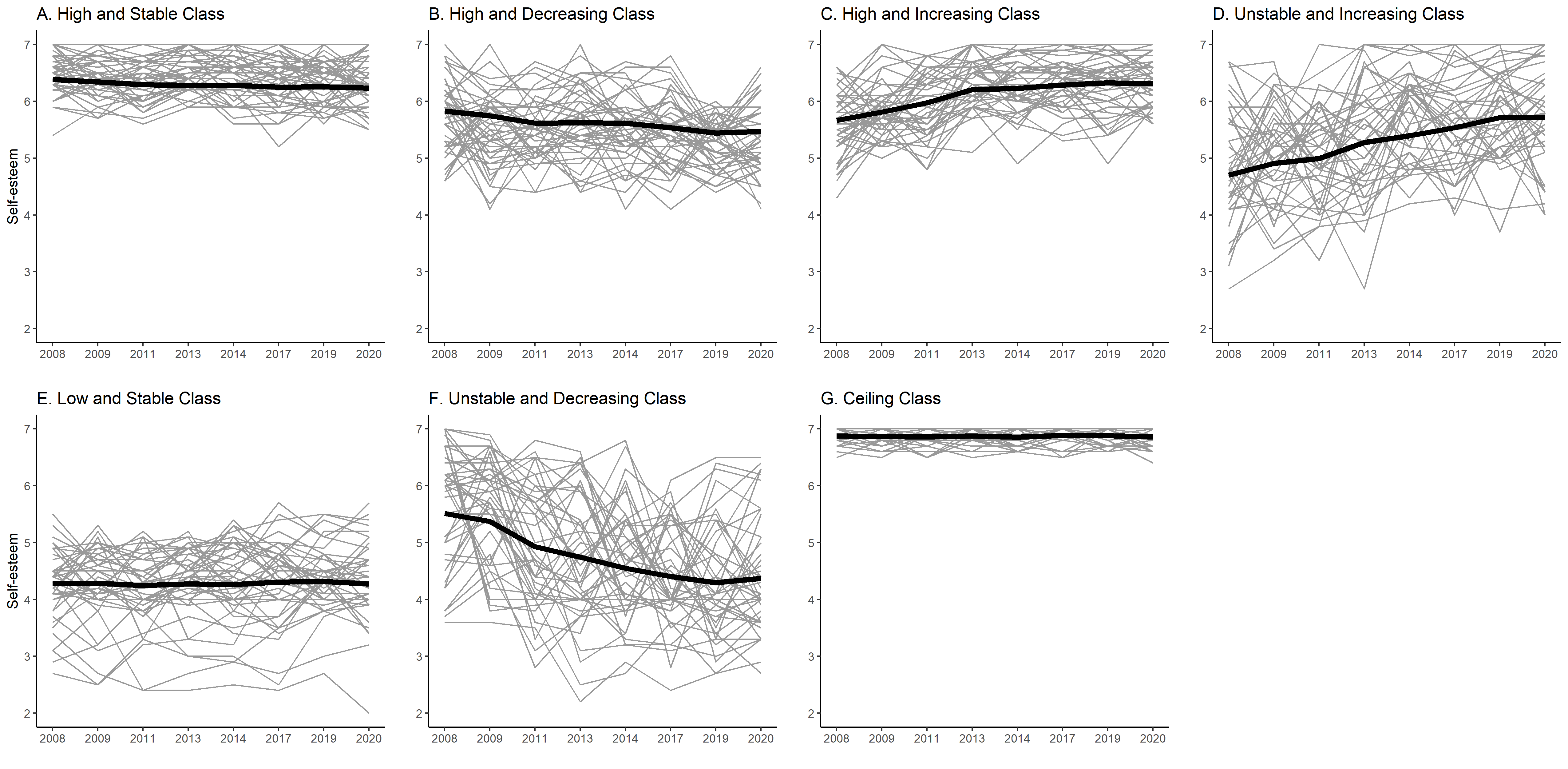


**Appendix.Figure 2**

*Class Parameters Plots of Sensitivity Analysis Using All Available Waves (Including the Waves With Few Responses)*


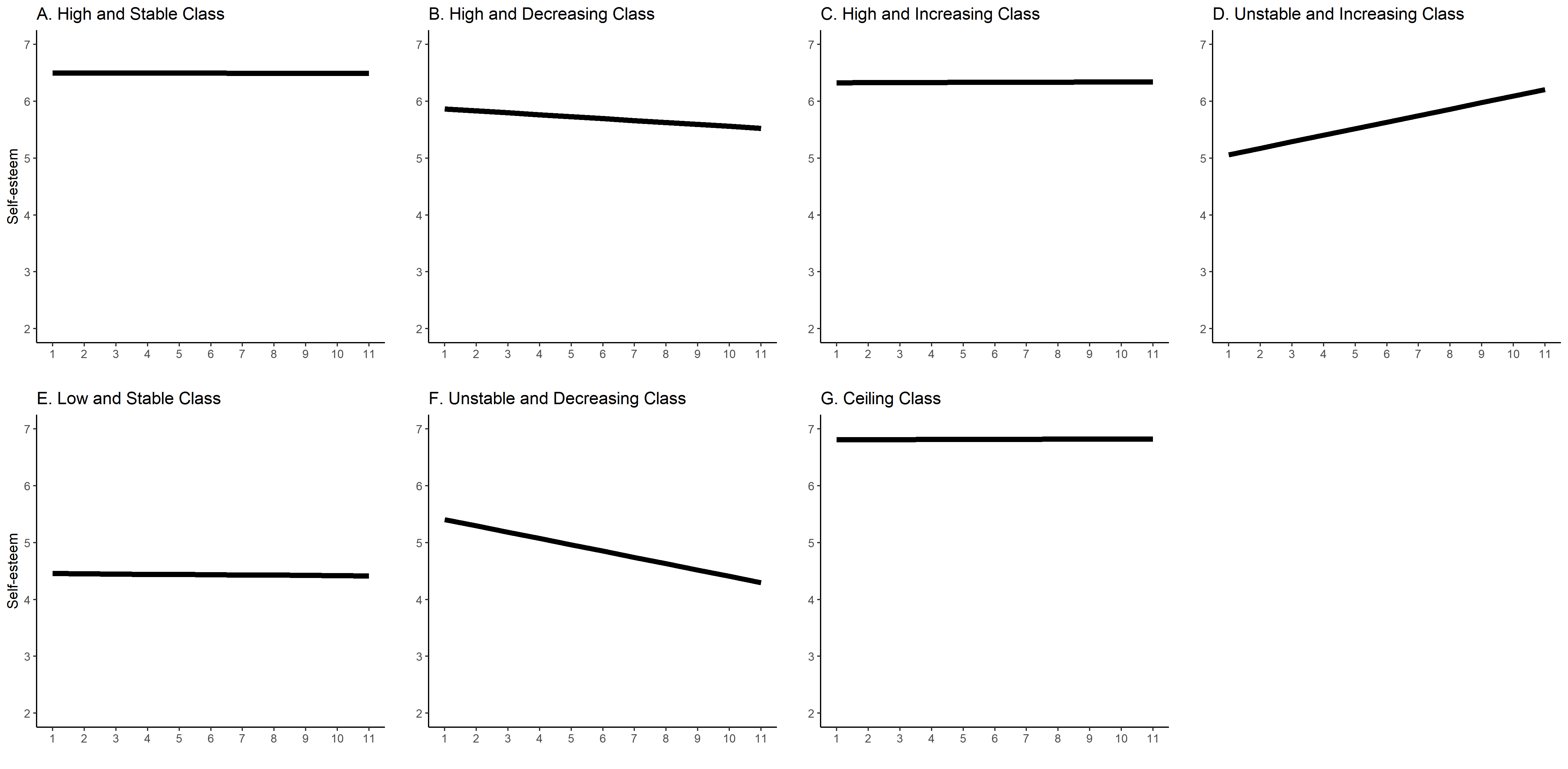

Supplement: Supplementary file 1 — Supplementary Material 1. [file 10608_2025_10604_MOESM1_ESM.docx]
